# Engineering *Halomonas bluephagenesis* TD01 as a Robust Chassis for the Sustainable Production of Hyaluronic Acid

**DOI:** 10.3390/biom16060846

**Published:** 2026-06-09

**Authors:** Ehab Marwan-Abdelbaset, Xiaoyun Lu, Dan Tan

**Affiliations:** 1 Key Laboratory of Biomedical Information Engineering of Ministry of Education, School of Life Science and Technology, Xi’an Jiaotong University, Xi’an 710049, China; ehab_emm@azhar.edu.eg (E.M.-A.); luxy05@xjtu.edu.cn (X.L.); 2 Department of Botany and Microbiology, Faculty of Science, Al-Azhar University, Nasr City, Cairo 11884, Egypt

**Keywords:** hyaluronic acid, *Halomonas bluephagenesis* TD01, Response Surface Methodology (RSM), molecular weight tuning, high hyaluronic acid molecular weight, haloalkaliphilic bacteria

## Abstract

This study evaluates the development of *Halomonas bluephagenesis* TD01 as a novel, sustainable microbial platform for the production of hyaluronic acid (HA). Three distinct hyaluronan synthase genes (*sezHasA* and *spHasA* "Class I" from the Streptococcal group and *pmHasA* "class II") were heterologously expressed and compared, with the Class II synthase from *Pasteurella multocida* (*pmHasA*) emerging as the superior variant in rich media 60-LBG, achieving significantly higher titers of 0.88 g/L and molecular weight (*M_w_*) of 1.15 MDa (Mega Daltons). Using a combination of Plackett–Burman design and Response Surface Methodology (RSM), the fermentation process was optimized, identifying initial pH, nitrogen source, and NaCl concentration as critical factors. These optimizations led to a maximum HA yield from 0.88 to 2.38 g/L (265% improvement) and *M_w_* from 1.15 to 9.67 MDa. Furthermore, the study demonstrates precise tuning of HA molecular weight, ranging from 2.04 MDa to 9.67 MDa in a modified medium (40LBG-Y), by modulating L-arabinose induction levels. The structural integrity of the purified HA was confirmed via ESI-MS and ^1^H-NMR. These findings establish *H. bluephagenesis* TD01 as a robust Next-Generation Industrial Biotechnology (NGIB) chassis for the scalable and customizable production of HA with a minimal cost and high-molecular-weight HA for medical applications.

## 1. Introduction

Hyaluronic acid (HA), also known as hyaluronan, is a linear polysaccharide consisting of repeating disaccharide units of GlcNAc (β1-3 D-N-acetyl glucosamine) and GlcA (β1-4 D-glucuronic acid) [1]. Due to its unique viscoelastic properties and remarkable water-retention capacity, HA performs essential structural, physiological, and biological functions in living organisms. These properties have enabled its widespread use in pharmaceutical, biomedical, and cosmetic industries [2,3]. The biological effects and specific applications of HA are dictated by its molecular weight (*M_w_*), which can range from less than 1000 Daltons to several million Daltons, as *M_w_* regulates critical physiological responses and receptor interactions [4,5].

Historically, HA has been commercially produced through extraction from rooster combs or via fermentation using pathogenic *Streptococcus* strains. As was reported with *S. zooepidemicus*, the highest HA production achieved was 2.825 g/L/4.5 L in a bioreactor with controlled pH (8.0) and medium containing molasses [6]. In another study, a mutant *S. zooepidemicus* strain in a semi-continuous fermentation process consisting of two-stage 3 L bioreactors was developed, in which 1.01 g/L/h productivity of hyaluronic acid was obtained; after recombinant hyaluronidase SzHYal was added into the second-stage bioreactor at 6 h, the values were 14.60 g/L, and 29.38 g/L, respectively [7]. However, neither method is ideal; extraction poses risks of avian allergen contamination, while *Streptococcus* fermentation carries the danger of exotoxin contamination. Furthermore, both methods often suffer from HA degradation during purification. To mitigate these safety concerns, industrial biotechnology has sought to engineer GRAS (Generally Recognized As Safe) platforms such as *Bacillus subtilis*, *Agrobacterium*, *E. coli* JM-109, *E. coli* K12 W3110, *Lactococcus lactis*, and *Corynebacterium glutamicum* for large-scale production [8,9,10,11]. Despite these advancements, the production costs of HA remain soaringly high [12,13]. Current bioprocesses are limited by high sterilization costs, heavy energy consumption, and a massive demand for fresh water, all while remaining vulnerable to microbial contamination [14].

To overcome these economic and technological hurdles, Next-Generation Industrial Biotechnology (NGIB) has emerged as a sustainable alternative. NGIB utilizes robust microbial chassis that thrive under harsh conditions, allowing for continuous fermentation under open, non-sterile environments [15,16]. A primary candidate for this approach is *Halomonas bluephagenesis* TD01, a rapidly growing haloalkaliphilic bacterium. *H. bluephagenesis* TD01 can grow in seawater under high salinity and high pH (NaCl 60 g/L, pH 9.0), effectively preventing contamination without the need for energy-intensive sterilization [15,16,17]. Crucially, this strain possesses intrinsic biosynthesis pathways for GlcNAc and GlcA; therefore, engineering it for HA production requires only the introduction of a hyaluronan synthase (HAS) enzyme to facilitate the binding and polymerization of these precursors [18,19].

The production of HA is a tightly regulated process where gene induction allows cells to adapt to environmental stressors and conserve energy [20,21]. The expression of the HasA gene, which encodes hyaluronan synthase, is influenced by several factors, including nutrient availability (nitrogen sources), temperature shifts (heat or cold shock), and environmental pH [22]. Additionally, high salt concentrations can induce osmotic stress responses that activate specific stress-related genes, while chemical inducers (such as nisin or arabinose) can be used to precisely trigger transcription [23,24,25]. Regulating the ratio of hyaluronan synthase to precursor sugar concentrations (such as UDP-GlcA) serves as an efficient strategy for controlling the resulting HA molecular weight. Higher expression levels of HasA typically correspond to higher *M_w_* HA, provided the precursor supply remains sufficient [26,27].

Because gene expression is highly sensitive to the surrounding environment, optimizing culture conditions is the most direct method for maximizing both HA yield and *M_w_*. Traditional “one-factor-at-a-time” optimization methods are often inadequate as they fail to account for the complex interactive effects between different variables [28]. To achieve a truly optimized process, statistical modeling is required. The Plackett–Burman design offers a novel way to screen and identify the most significant parameters from a large pool of variables [29]. Subsequently, Response Surface Methodology (RSM) serves as a reliable tool to assess the independent and interactive effects of factors such as nitrogen sources, NaCl concentration, pH, temperature, and agitation speed on HA production [30].

**Figure 1:**
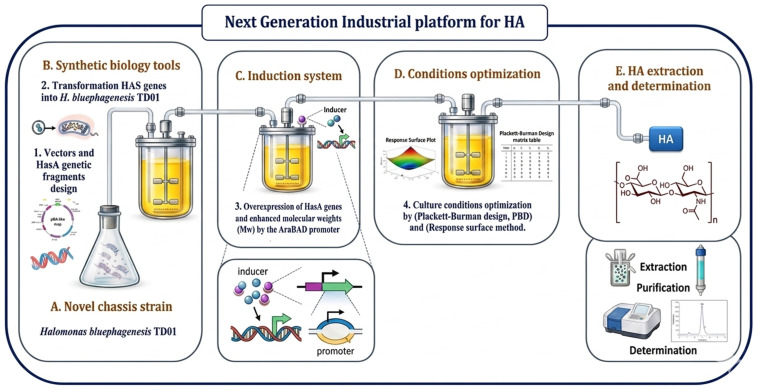
Integrated flowchart for HA production using the alkalohalophilic platform *H. bluephagenesis* TD01 for hyaluronic acid (HA) production.

This study represents the first report on the development of *H. bluephagenesis* TD01 as a platform for HA production through the cloning of different Class I and Class II HAS genes. Specifically, we compare three distinct HAS enzymes: pmHAS from *Pasteurella multocida* (972 amino acids), and the smaller sezHAS and spHAS from *Streptococcus equi* subsp. *zooepidemicus* and *S. pyogenes* (419 and 417 amino acids, respectively) [31]. By engineering this industrial host, we aim to maximize HA yield and tailor its molecular weight while significantly reducing production costs. This research provides a comprehensive comparative analysis of *HasA* gene expression in *H. bluephagenesis* TD01, exploring the relationship between environmental conditions, gene expression levels, and the resulting physiological characteristics of the synthesized hyaluronan. Through the integration of molecular engineering and statistical optimization, this work establishes a path toward low-cost, sustainable, and high-efficacy HA production (Figure 1).

## 2. Materials and Methods

### 2.1. Bacterial Strains, Plasmids, and Culture Conditions

The bacterial strains and plasmids used or developed in this study are summarized in Table 1. *H. bluephagenesis* TD01, a haloalkaliphilic bacterium originally isolated from Aydingol Lake in Xinjiang Province, China, served as the primary host for HA production. Escherichia coli DH5α and S17-1 were employed for plasmid construction and as vector donor strains during conjugation, respectively. All *E. coli* strains were cultured in LB medium (10 g/L tryptone, 5 g/L yeast extract, and 10 g/L NaCl) or LB-20 medium (supplemented with 20 g/L NaCl). *H. bluephagenesis* TD01 and its derivatives were cultivated in 60-LB medium (10 g/L tryptone, 5 g/L yeast extract, and 60 g/L NaCl). For hyaluronic acid (HA) production, two specific media were utilized: 60-MMG mineral medium, prepared according to [16], and 60-LBG medium consisting of LB-60 supplemented with 20 g/L glucose. Unless otherwise stated, the pH for *Halomonas* cultures was adjusted to 9.0. When necessary, chloramphenicol was added at a final concentration of 25 mg/L (or 25 µg/mL) for selection. All chemicals and reagents were sourced from Sinopharm Chemical Reagent Co., Shanghai, China, and Sigma-Aldrich, St. Louis, MO, USA.

### 2.2. Construction of Recombinant Strains

#### 2.2.1. Plasmid Construction

The plasmids utilized in this study are detailed in Table 1. All molecular cloning procedures, including DNA purification and high-quality plasmid isolation, were performed using kits from Qiagen (Shanghai, China) following the manufacturer’s protocols. Restriction endonucleases and DNA modification enzymes were obtained from New England Biolabs (Ipswich, MA, USA). A novel expression vector, designated pMCS-eSD-sfGFP-backbone, was successfully engineered to facilitate HA production. This backbone incorporates the following essential genetic elements: the HasA gene, encoding the hyaluronan synthase protein; a Multiple Cloning Site (MCS) with strategically placed restriction enzyme sites for precise HasA sequence insertion; the ParaC-araBAD promoter system for inducible control; the pMABY-KS origin of replication and a chloramphenicol resistance (*Cm*^R^) marker for selection; and the origin of transfer (oriT), enabling mobilization during conjugation from *E. coli* to *H. bluephagenesis* TD01.

#### 2.2.2. Conjugation into *H. bluephagenesis* TD01

The transfer of recombinant plasmids from *E. coli* S17-1 to the *H. bluephagenesis* TD01 recipient strain was performed via biparental conjugation. *E. coli* S17-1 donor strains, harboring the target plasmids, were cultured in LB medium, while the recipient DNA-host (TD01) was grown in 60-LB medium, both supplemented with the appropriate selective antibiotics. Overnight cultures of both the donor and recipient were used to inoculate fresh LB and 60-LB media (1% *v*/*v*), respectively, and were incubated for 6 h to reach the exponential growth phase. Subsequently, 1 mL of each culture was harvested by centrifugation (6000× *g* for 2 min), washed, and resuspended in 50 µL of their respective media.

The concentrated donor and recipient cells were then mixed and spotted onto 20-LB agar plates (containing 20 g/L NaCl) to facilitate cell-to-cell contact. Following overnight incubation at 37 °C, the resulting bacterial “moss” was scraped from the plate and resuspended in 100 µL of 60-LB medium. Finally, the suspension was spread onto 60-LB agar plates supplemented with the appropriate antibiotics for selection. These plates were incubated at 37 °C for 24–96 h until recombinant colonies were clearly visible.

#### 2.2.3. Confirmation of Transconjugants by Colony PCR and DNA Sequencing

To verify the successful carriage of the pMCSeSD-HasA plasmids, five to eight colonies were randomly selected from the transconjugant selective plates for screening. Colony PCR was performed using specific primers designed for the HasA gene (Part A of Supplementary Materials). Individual colonies were sampled using a sterilized toothpick and resuspended directly into a 25 μL PCR reaction mixture. Each reaction contained: 2.5 μL 10× PCR buffer (MgCl_2_-free), 2.5 μL MgCl_2_ (50 mM), 2.0 μL dNTPs (2.5 mM each), 0.25 μL Taq DNA polymerase (New England BioLabs, Woburn, MA, USA), 1.0 μL each of the forward and reverse primers (25 pmol/μL), and sterile distilled water to a final volume of 25 μL. DNA amplification was carried out in a thermocycler (Eppendorf™ Model #AG 22331, Hamburg, Germany) using the following parameters: an initial denaturation at 95 °C for 10 min; followed by 30 cycles of denaturation at 95 °C for 1 min, annealing at 55 °C for 1 min, and extension at 72 °C for 1.5 min; with a final extension at 72 °C for 5 min. The resulting PCR products were visualized via 1.5% (*w*/*v*) agarose gel electrophoresis to confirm the presence of the predicted 1254 bp *sezHasA*, 1257 bp *spHasA*, and 2916 bp *pmHasA* bands corresponding to the HasA marker. Finally, the identity of the transconjugants was further validated through Sanger sequencing (Thermo Fisher Scientific, Waltham, MA, USA).

### 2.3. Growth Curve Characterization

To monitor bacterial growth, the recombinant strains were cultivated in both 60-LBG and 60-MMG media. Once the cultures reached an early exponential phase (OD_600_ of 0.4–0.5, approximately 4 h post-inoculation), bacterial growth was monitored over 24 h using a microplate reader (Thermo MULTISKAN Spectrum, Thermo Fisher Scientific, USA). Aliquots of 200 μL from each culture were transferred to microplate wells in triplicate to ensure statistical reliability [33]. The plates were sealed with pre-processed lids and maintained under continuous rotary shaking at 200 rpm and 30 °C (LYZ-2102C Shaking Incubator, Longyue, China). The *OD*_600 nm_ was recorded every 2 h, with sterile 60-LBG and 60-MMG media serving as blank controls. After subtracting the blank values, the average *OD*_600 nm_ for each time point was used to construct the growth curves. To determine the cell dry weight (CDW), the biomass was lyophilized until a constant weight was achieved [34].

### 2.4. Shake Flask Cultivation and Verification of HAS Activity

Following the successful conjugation and PCR verification of the recombinant plasmids into *H. bluephagenesis* TD01, three experimental strains were established: TD01-pMCSeSD-*pmHasA*, TD01-pMCSeSD-seHasA, and TD01-pMCSeSD-*spHasA*. Each strain harbors the pMCSeSD-*Cm*^R^ plasmid containing its respective HasA gene. Wild-type *H. bluephagenesis* TD01 was utilized as the negative control.

To prepare the primary seed culture, a single colony from a freshly streaked agar plate was inoculated into a shake flask (HZQ-F160, HDL, Harbin, China) containing 60-LB medium and incubated at 37 °C and 200 rpm for 12–14 h. Subsequently, this primary culture was used to inoculate fresh 60-LB medium at a 1% (*v*/*v*) ratio and grown for 8–10 h to produce the secondary seed culture. For HA production, 2.5 mL of the secondary seed culture was inoculated into 50 mL of 60-LB medium (5% *v*/*v* inoculum) supplemented with 25 µg/mL chloramphenicol. The production medium (pH 8.5 ± 0.5, adjusted using 5 M NaOH) consisted of 10 g/L tryptone, 5 g/L yeast extract, and 60 g/L NaCl. The flasks were incubated on a rotary shaker at 30 °C and 200 rpm for 72 h. All experimental groups were performed in triplicate to ensure statistical accuracy.

After 48 h of cultivation, the broth was harvested to determine HA yield and molecular weight (*M_w_*). The cultures were centrifuged at 10,000× *g*, and the resulting cell pellets were washed twice with distilled water to remove residual media components. To determine the cell dry weight (CDW), the biomass was lyophilized until a constant weight was achieved. The supernatant was retained for subsequent HA quantification and *M_w_* analysis. Hyaluronic acid was extracted and purified following the protocol described by Brown et al. [35] with minor modifications. The procedure was carried out as follows:

### 2.5. Extraction and Purification of HA

To release the HA from the bacterial capsules, 50 μL of 10% (*w*/*v*) sodium dodecyl sulfate (SDS) was added to the culture, followed by incubation at room temperature for 10–15 min with intermittent shaking. Subsequently, 2 mL of 10% (*w*/*v*) cetyltrimethylammonium bromide (CTAB) was added to the Erlenmeyer flasks. The mixture was incubated at 10 °C for 1 h and then centrifuged at 4000 rpm for 20 min to collect the HA-CTAB complex pellet. The resulting pellets were solubilized in 10 mL of 2 M CaCl_2_ and maintained at 4 °C for 6 h. Following centrifugation (2200× *g*, 20 min), the supernatant was collected, and two volumes of absolute ethanol were added. The solution was incubated at 4 °C for 1 h to precipitate the HA. The precipitate was recovered by centrifugation (2200× *g*, 20 min) and redissolved in 10 mL of bi-distilled water overnight at 4 °C with gentle agitation.

The solution was centrifuged again (2200× *g*, 20 min), and the supernatant was mixed with an equal volume of 1% (*w*/*v*) NaCl. The HA was re-precipitated using two volumes of absolute ethanol and centrifuged. The final pellet was solubilized in 5 mL of bi-distilled water overnight at 4 °C. This centrifugation and solubilization cycle were repeated to ensure high purity. The final supernatant was filtered through 0.45 μm nitrocellulose mixed ester syringe filters and stored at 4 °C for further analysis. Analytically pure HA (Sigma-Aldrich, St. Louis, MO, USA) was used as a standard for quantification.

### 2.6. HA Characterization

#### 2.6.1. Identification by Mass Spectrometry (MS)

For structural identification, HA standard and unknown samples were subjected to enzymatic cleavage to produce disaccharide units. A phosphate-buffered saline (PBS) solution (pH 7.0) was prepared and filtered through a 0.45 μm syringe filter. Hyaluronidase was dissolved in PBS to a final concentration of 10 U/mL. The enzymatic reaction was initiated by adding 12 U/mL of hyaluronidase to the HA samples, followed by incubation at 37 °C and 200 rpm for 48 h. The reaction was terminated by heating the mixture to 70 °C for 25 min. Before MS analysis, the treated samples were filtered through a 0.22 μm syringe filter and stored at 4 °C. Mass spectrometry was performed using a Thermo LCQ Deca system (Thermo Fisher Scientific, Waltham, MA, USA). The resulting disaccharides were dissolved in 50% methanol and analyzed in negative ionization mode with the following parameters: spray voltage, 5 kV; capillary temperature, 275 °C.

#### 2.6.2. Nuclear Magnetic Resonance (^1^H-NMR) Spectroscopy

The composition and purity of the extracted HA and the commercial standard were analyzed using ^1^H-NMR spectroscopy. Before analysis, all samples were lyophilized to remove residual moisture. The samples were prepared by dispersing the resulting gels in deuterium oxide (D_2_O) at a concentration of 1 mg/mL. ^1^H-NMR spectra were recorded at 25 °C using a Bruker Avance™ 400 MHz (or 500 MHz) spectrometer (Billerica, MA, USA). The acquisition parameters included the following: scans, 256; acquisition time, 2.56 s; relaxation delay, 1.0 s; pulse angle, 90°. Chemical shifts (δ) are reported in parts per million (ppm) and were referenced to the residual solvent signal of D_2_O at 4.79 ppm.

### 2.7. Induction of HasA Gene Expression and Culture Optimization

**Figure 2:**
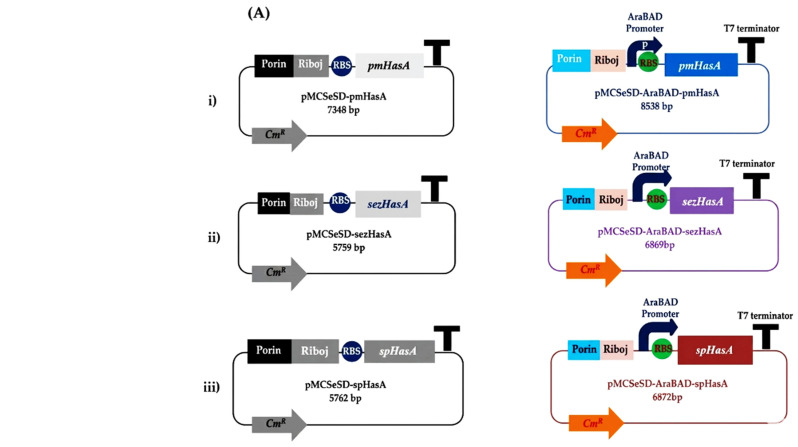

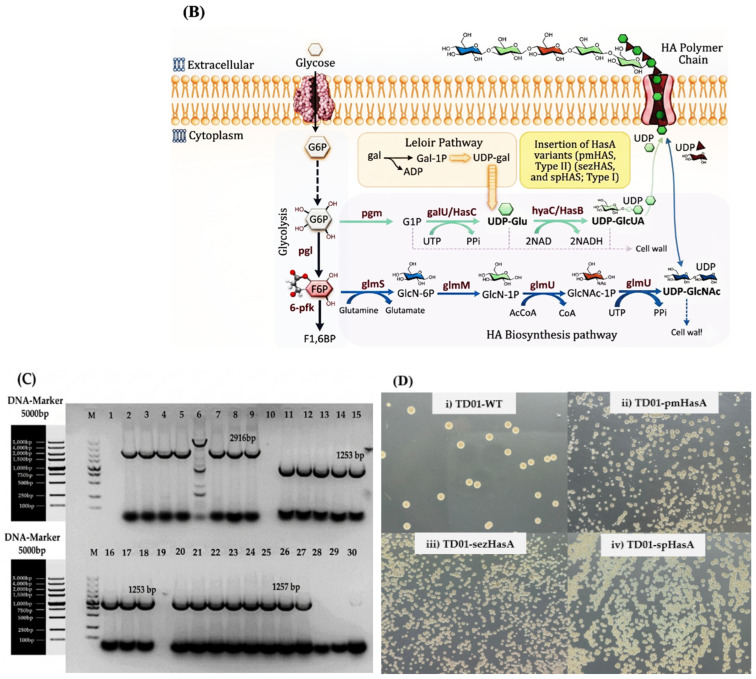
Molecular construction and phenotypic validation of recombinant *H. bluephagenesis* TD01. (**A**) Schematic maps of recombinant expression vectors: The genetic architecture of the plasmids designed for hyaluronic acid (HA) production illustrates the organization of various hyaluronan synthase (HasA) genes, including configurations with and without inducible promoters, and highlights the required regulatory elements. (**B**) Engineering *H. bluephagenesis* TD01 for HA biosynthesis from glucose. HA chains are elongated by HA synthase, reportedly by HasAs (*pmHAS*, *sezHAS*, and *spHAS* in this study), from two building blocks: UDPGlcA (uridine diphosphate glucuronate) and UDP-GlcNAc (uridine diphosphate N-acetylglucosamine). Pgm, phosphoglucomutase; GalU, glucose-1-phosphate uridylyltransferase; hyaC, UDP-glucose 6-dehydrogenase; Pgi, glucose-6-phosphate isomerase; GlmS, L-glutamine-D-fructose-6-phosphate aminotransferase; GlmM, phosphoglucosamine mutase; GlmU, UDP-N-acetylglucosamine pyrophosphorylase/Glucosamine-1-phosphate N-acetyltransferase. (**C**) Molecular confirmation of HasA integration: verification of the HasA cassettes within *H. bluephagenesis* TD01 transconjugants was performed via colony PCR and 1.5% agarose gel electrophoresis. M: DNA ladder (5000 bp). Lanes 2–9: TD01-pMCSeSD_AraBAD_*pmHasA* (expected amplicon: 2916 bp). Lane 6 represents an unsuccessful transconjugant. Lanes 11–18: TD01-pMCSeSD_*AraBAD*_*sezHasA* (expected amplicon: 1253 bp). Lanes 20–27: TD01-pMCSeSD_AraBAD_*spHasA* (expected amplicon: 1257 bp). Lanes 28–30: wild-type (WT) *H. bluephagenesis* TD01, serving as the negative control. (**D**) Comparative colony morphological appearance of wild-type and recombinant colonies after 24 h of incubation at 30 °C on 60-LB agar. The recombinant strains exhibit a distinct mucoid morphology characterized by a glossy, viscous, and elevated surface consistent with the successful synthesis of extracellular hyaluronic acid. In contrast, the wild-type colonies appear more defined with a matte, non-reflective surface, indicating an absence of polysaccharide overproduction.

The expression vectors, containing the various HasA genes under the control of the P_BAD_ promoter (Figure 2A), were introduced into *H. bluephagenesis* TD01 via electroporation. Successful transformants were confirmed through colony PCR using gene-specific primers (Supplementary Material Table S1). To optimize the inducible system, recombinant strains were grown in selective media until reaching an early exponential phase (*OD*_600 nm_ of 0.4 to 0.5, approximately 4 h post-inoculation). For initial concentration screening, L-arabinose was added at varying final concentrations: 0.05%, 0.1%, 0.2%, 0.4%, 0.6%, 0.8%, and 1% (*w*/*v*). Unless otherwise specified, a standard concentration of 0.2% L-arabinose was used for subsequent induction experiments. Cultures were incubated in 50 mL shake flasks (initial inoculum of 2.5 mL seed culture) at 30 °C and 200 rpm for 72 h under chloramphenicol selection (25 µg/mL). The pH was maintained between 8.5 and 9.0 by the addition of 5 M NaOH [23,36]. All experimental groups were performed in triplicate.

Induction experiments were conducted in two primary media, both supplemented with 2% (*w*/*v*) glucose as the carbon source [37]: 60-LBG (rich medium) was composed of (g/L) 60 NaCl, 20 glucose, 5 yeast extract, and 10 tryptone. 60-MMG (mineral medium) was composed of (g/L) 60 NaCl, 20 glucose, 5 yeast extract, 1 NH_4_Cl, 0.2 MgSO_4_, 9.65 Na_2_HPO_4_ · 12H_2_O, and 1.5 KH_2_PO_4_. This was supplemented with 10 mL/L Trace Element Solution I and 1 mL/L Trace Element Solution II. The trace element solutions were prepared as follows. Solution I (g/L): 5 Fe (III)-NH_4_-citrate, 2 CaCl_2_, and 1 M HCl. Solution II (mg/L): 100 ZnSO_4_.7H_2_O, 30 MnCl_2_.4H_2_O, 300 H_3_BO_3_, 200 CoCl_2_.6H_2_O, 10 CuSO_4_.5H_2_O, 20 NiCl_2_.6H_2_O, 30 Na_2_MoO_4_.2H_2_O, and 1 M HCl. Following the 72 h incubation, the bacteria were harvested by centrifugation at 10,000× *g*. The resulting cell pellets were washed once with distilled water and lyophilized to determine the cell dry weight (CDW). The supernatant and processed biomass were further analyzed for HA yield determination.

### 2.8. Quantification of HA

#### 2.8.1. Turbidimetric Method

The concentration of produced HA was determined using a turbidimetric assay as described by Oueslati et al. [38]. The method relies on the formation of an insoluble complex between the negatively charged HA and the cationic surfactant cetyltrimethylammonium bromide (CTAB). Two primary reagents were prepared for the assay: (i) Reagent 1: 2% (*w*/*v*) NaOH (2 g NaOH dissolved in 100 mL distilled water). (ii) Reagent 2: 2.5% (*w*/*v*) CTAB solution (2.5 g CTAB dissolved in 100 mL of Reagent 1).

The quantification procedure was performed as follows: A 1 mL sample was transferred to a test tube and incubated at 37 °C for 15 min. A total of 2 mL of Reagent 2 was added to the sample. The mixture was incubated at 37 °C for an additional 10 min, with vigorous shaking for 10 s at both the start and the end of the incubation period to ensure uniform turbidity. The resulting turbid solution was transferred to an ELISA microplate. The absorbance was measured at 600 nm using a microplate reader (Thermo MULTISKAN Spectrum, Thermo Fisher Scientific, USA). Sterile medium processed under the same conditions served as the blank. The average optical density (*OD*_600 nm_) for each sample was calculated, and the HA concentration was quantified by referencing a standard curve generated with analytically pure HA.

#### 2.8.2. Determination of HA Molecular Weight (*M_w_*)

The molecular weight of purified HA produced by the recombinant strains TD01-pMCSeSD-*pmHasA*, TD01-pMCSeSD-*sezHasA*, and TD01-pMCSeSD-*spHasA* without L-arabinose induction was determined using Aqueous Gel Permeation Chromatography (GPC). Intracellular and extracellular HA were recovered from lyophilized cells through a sequence of chloroform extraction and cold ethanol precipitation [39]. The resulting purified HA was dissolved in the mobile phase at a final concentration of 10 mg/mL.

Analysis was performed using a GPC system (SHIMADZU, Kyoto, Japan) equipped with a Refractive Index Detector (RID). The chromatographic separation was achieved using a high-performance column system (SB-807 HQ and SB-806M HQ or SHIMADZUGPC-804C). The experimental conditions were maintained as follows. Mobile Phase: 0.1 M NaNO_3_ solution. Flow Rate: 0.5 mL/min. Injection Volume: 100 µL. Temperature: Ambient. Molecular weight was calculated using a calibration curve generated with Poly (ethylene oxide) (PEO) standards. Additionally, a commercial hyaluronic acid sodium salt (880 kDa to 1.8 MDa; Sigma-Aldrich, St. Louis, MO, USA) was utilized as a benchmark standard to verify the accuracy of the GPC analysis [40].

### 2.9. Optimization of HA Production

#### 2.9.1. Preliminary Screening via One-Factor-at-a-Time (OFAT)

To identify the critical environmental and nutritional parameters influencing HasA gene expression and HA biosynthesis, a one-factor-at-a-time (OFAT) approach was initially employed. This classical method allowed for the systematic screening of individual variables to determine the conditions resulting in the highest HA yield. Following the comparative analysis of the three recombinant strains, *H. bluephagenesis* TD01-pMCSeSD-*AraBAD-pmHasA* was selected as the optimal candidate for further study due to its superior HA yield and molecular weight (*M_w_*) profile. This classical optimization method allowed for the systematic screening of individual variables, including carbon and nitrogen sources, salinity, and pH, to determine their independent effects on HA production.

##### The Effect of Nitrogen Sources, Salinity, and Initial pH (OFAT)

To optimize HA production and identify the factors most significantly affecting HasA gene expression, we undertook evaluation of nitrogen sources, salinity and pH as environmental factors: (i) The recombinant strain, TD01-pMCSeSD-*AraBAD-pmHasA*, was tested using various nitrogen sources to determine their impact on biosynthesis. The following sources were evaluated at a final concentration of 15 g/L: organic sources: tryptone, yeast extract, casein, and urea; inorganic sources: ammonium chloride (NH_4_Cl) and ammonium sulfate (NH_4_)_2_SO_4_; complex source: a combination of 10 g/L tryptone and 5 g/L yeast extract [41]. (ii) To assess the haloalkaliphilic requirements of the host, NaCl concentrations were tested at 2%, 4%, 6%, 8%, 10%, and 12% (*w*/*v*). (iii) Initial pH ranged from 5.0 to 10.0 (5.0, 6.0, 7.0, 8.0, 9.0, and 10.0). The pH was precisely adjusted using 1 N HCl or 1 N NaOH. The experimental cultures were inoculated into both 60-LBG media and incubated at 30 °C with a rotary shaking speed of 200 rpm for 72 h. All experiments were performed in triplicate. The optimal nitrogen source, salinity level, and initial pH were selected based on the maximum observed HA yield to serve as the baseline for subsequent Plackett–Burman design (PBD) and Response Surface Methodology (RSM) design.

##### The Effect of Temperature, Incubation Time, and Agitation Rate

To further refine the cultivation parameters, the impact of physical variables on both bacterial growth and HA biosynthesis was systematically evaluated. Initial baseline studies were conducted under batch fermentation conditions at 30 °C, 200 rpm, and pH 9.0. Subsequently, the following ranges were screened to identify optimal conditions for maximizing HA yield. Temperature: 25 °C to 40 °C. Agitation Rate: 100 rpm to 250 rpm. Incubation Time: 24 to 72 h. The selection of these specific ranges for pH (5–10), temperature, and agitation was informed by established batch fermentation protocols for halophilic biopolymers [42]. All trials were monitored to determine the correlation between metabolic activity and the physical environment, ensuring the stability of the recombinant strain throughout the production cycle.

#### 2.9.2. Statistical Optimization of HA Production via Design of Experiments (DOE)

While the one-factor-at-a-time (OFAT) method provides a baseline for optimal growth conditions, it does not account for the complex interactions among bioprocess variables. To address this and maximize hyaluronic acid (HA) production in *H. bluephagenesis* TD01-pMCSeSD-*araBAD-pmHasA*, a structured design of experiments (DOE) approach was implemented. DOE enables the simultaneous evaluation of multiple factors to determine their individual and interactive effects on the target response, in this case, HA yield. Specifically, this multi-stage experimental framework was developed to account for potential synergistic or antagonistic interactions between key cultivation parameters, such as pH, NaCl concentration, temperature, and nutrient concentrations.

Therefore, to achieve a more robust and precise optimization, the process utilized a series of advanced statistical designs. First, a Plackett–Burman design (PBD) was used to screen a broad range of factors and identify those with the most significant impact on HA yield. Subsequently, Response Surface Methodology (RSM), specifically central composite design, was used to model the interactive effects of the significant factors identified by the PBD. This final stage determined the absolute optimal values for maximizing both HA yield and molecular weight.

#### 2.9.3. Screening of Significant Variables via Plackett–Burman Design (PBD)

Following the identification of optimal nitrogen sources, salinity levels, and physical parameters through OFAT, a Plackett–Burman design (PBD) was employed to screen for variables with a statistically significant impact on HA production. This design allows for the evaluation of a large number of factors with a minimum number of experimental runs to identify the primary drivers of the biosynthetic process. A total of eight independent variables were evaluated, each tested at two levels: high (+1) and low (−1). The factors screened included: nutritional factors (yeast extract concentration and the combination of tryptone + yeast extract); environmental factors (NaCl concentration (2% and 6% *w*/*v*) and initial pH 7.0 and 9.0); and physical factors (agitation speed 200 and 250 rpm, incubation time 48 and 72 h, and incubation temperature 25 and 30 °C). To ensure statistical robustness and estimate experimental error, three dummy variables were included in the design matrix (these are unassigned factors that do not represent any physical component of the media or environment). It was included to estimate the standard error of the experiment and to calculate the statistical significance (*p*-values) of the 8 real variables. All experimental runs were performed in triplicate. The PBD results were analyzed using Minitab (Version 21.1.0) and Design-Expert (Version 10.0.1, Stat-Ease Corp., Minneapolis, MN, USA). The significance of each variable was determined using Analysis of Variance (ANOVA), and those with a *p*-value < 0.05 were selected for further optimization through Response Surface Methodology (RSM).

#### 2.9.4. Statistical Optimization via Response Surface Methodology (RSM)

To optimize the levels of the significant factors identified during the screening phase, a central composite design (CCD) was implemented using Design-Expert (Version 10.0.1, Stat-Ease Corp., USA). This Response Surface Methodology (RSM) was utilized to model the interactive effects of the variables and to determine the precise coordinates for maximum HA yield.

Based on the statistical significance indicated by the PBD Pareto chart, three critical independent factors were selected for further optimization: Factor A, initial pH; Factor B, nitrogen source concentration (yeast extract/tryptone); Factor C, NaCl concentration. A 23-factorial design, augmented with six axial (star) points and six central points, resulted in a total of 20 experimental trials. Each factor was evaluated at five coded levels (−α, −1, 0, +1, and +α) to estimate the curvature of the response surface. The experimental data were fitted to a second-order polynomial equation to correlate the relationship between the independent variables and the HA yield (Y):

Y = ß_0_ + ∑ß_i_ X_i_ + ∑ß_ii_ Xi_2_ + ∑ß_ij_ X_i_X_j_ (1)

where Y is the predicted response (HA yield). ß_0_ is the intercept, and β_i_, β_ii_, and β_ij_ are the linear, quadratic, and interaction coefficients, respectively. The statistical significance of the model was validated using Analysis of Variance (ANOVA), and the quality of the fit was expressed by the coefficient of determination (R^2^).

### 2.10. Impact of L-Arabinose Concentration on HA Molecular Weight

After optimizing the culture medium and conditions and establishing the best parameters for HA production, to evaluate the influence of induction intensity on HAS activity and the resulting HA molecular weight (*M_w_*), a series of experiments were conducted using varied concentrations of the inducer. *H. bluephagenesis* TD01 harboring the pMCSeSD-*araBAD*-*pmHasA* construct was inoculated with 2.5 mL of seed culture into 50 mL of modified 40-LBG-Y medium with yeast extract only as a nitrogen source. To ensure optimal polymer chain extension, the cultures were incubated at a reduced temperature of 25 °C and an agitation rate of 250 rpm, with the initial pH adjusted to 8.0. Induction was initiated once the cultures reached an *OD*_600 nm_ of approximately 0.5, typically after 4 h of growth. L-arabinose was added at various final concentrations to assess its effect on the metabolic flux toward HA: inducer concentrations were 0.05%, 0.2%, 0.4%, 0.6%, 0.8%, and 1.0% *w*/*v*.

The fermentation was maintained for a total of 72 h to allow for the accumulation of high-molecular-weight HA. All experimental groups were performed in triplicate. Following incubation, the HA was harvested and purified as described in Section 2.5, and the molecular weight was determined via GPC analysis (as detailed in Section 2.8.2) to establish the correlation between inducer concentration and polymer chain length.

### 2.11. Statistical Analysis

All experiments were performed at least in triplicate, and results were expressed as the mean ± standard deviation. Based on the data structure, statistical analysis was performed using Student’s *t*-test (unpaired, two-tailed), one-way ANOVA, or two-way ANOVA with Bonferroni as a post-test in GraphPad Prism (version 8.0) software. Statistical significance was denoted as follows by *p* values: * *p* < 0.05; ** *p* < 0.01; *** *p* < 0.001; ns—non-significant.

## 3. Results

### 3.1. Construction and Confirmation of Recombinant H. bluephagenesis TD01 Strains

To establish *H. bluephagenesis* TD01 as a robust microbial platform for hyaluronic acid (HA) biosynthesis, three distinct expression vectors were engineered to harbor diverse hyaluronan synthase (HasA) genes.

#### 3.1.1. Genetic Architecture of Expression Vectors

As illustrated in Figure 2A, the plasmids pMCSeSD-*AraBAD-pmHasA* (8538 bp), pMCSeSD-*AraBAD*-*sezHasA* (6869 bp), and pMCSeSD-AraBAD-*spHasA* (6872 bp) were successfully constructed using the pMCSeSD-sfGFP backbone. Each vector utilizes the L-arabinose-inducible P_araBAD_ promoter system to provide tunable control over enzyme expression. The synthetic architecture of these cassettes was optimized for stability and high-level translation in the haloalkaliphilic host: a Porin-RiboJ insulator sequence was included to stabilize the mRNA transcript. A high-efficiency ribosome binding site (RBS) was positioned upstream of the HasA variants (*pmHasA* from *P. multocida*, *sezHasA* from *S. equi* subsp. *zooepidemicus*, and *spHasA* from *S. pyogenes*). A T7 terminator was placed downstream to ensure precise transcriptional termination. All constructs incorporate a chloramphenicol resistance marker (*Cm*^R^) for stable plasmid maintenance.

#### 3.1.2. Validation of Transconjugants via Colony PCR

Following the conjugation of the recombinant plasmids into *H. bluephagenesis* TD01, the presence of the expression cassettes was validated through colony PCR. As shown in Figure 2B,C, successful transconjugants were identified by the amplification of DNA bands corresponding to the specific HasA gene sizes. TD01-*pmHasA* amplicons appeared at the expected size of 2916 bp. TD01-*sezHasA* and TD01-*spHasA* yielded target bands at 1253 bp and 1257 bp, respectively. The screening indicated high transformation efficiency; for instance, seven out of eight selected colonies for the *pmHasA* variant were positive, with only one colony (Lane 6) failing to show the target band (Figure 2D). As expected, no amplification was observed in the wild-type (WT) control lanes, confirming both primer specificity and the successful integration of the synthetic HA-production machinery into the halophilic chassis.

### 3.2. Growth Characterization of Recombinant H. bluephagenesis TD01

Optimizing cultivation conditions is pivotal for achieving high biocatalytic conversion rates and maximizing HA yields. The initial phase of this study focused on evaluating the physiological impact of harboring various expression plasmids on the host chassis. The growth profiles of recombinant *H. bluephagenesis* TD01 strains carrying different *HasA* variants were characterized in both defined mineral (60-MMG) and rich (60-LBG) media, using the wild-type (TD01-WT) as a control. All cultures were maintained at an inoculum size of 5% (*v*/*v*), pH 9.0, 30 °C, and an agitation speed of 200 rpm.

#### 3.2.1. Growth Kinetics in Defined Mineral Medium (60-MMG)

**Figure 3:**
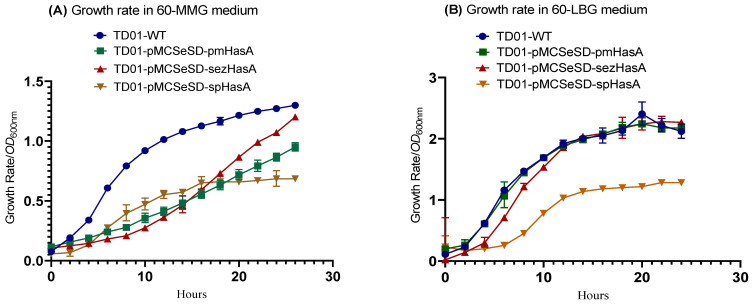
Growth kinetics for recombinant *H. bluephagenesis* TD01, which carries various expression of HasA gene plasmids in (**A**) 60-MMG medium and (**B**) 60-LBG medium. All data points are presented as the mean of triplicate experiments, with error bars indicating the standard deviation. The significant divergence of the *spHasA* growth curve reflects the varying degrees of metabolic stress imposed on the *Halomonas* chassis by different hyaluronan synthase variants on 60-LBG and 60-MMG, illustrating the increased sensitivity of the recombinant strains to metabolic stress when cultivated in a mineral-based environment compared to rich media.

To assess the robustness of the engineered strains under nutrient-limited, industrially relevant conditions, growth was monitored in 60-MMG. As illustrated in Figure 3A, the growth profiles in the mineral medium were markedly different from those typically observed in nutrient-rich environments. While the TD01-WT reached a maximum *OD*_600 nm_ (1.3), all three recombinant strains exhibited significantly reduced biomass accumulation. This divergence highlights a pronounced metabolic burden imposed by the synthetic HA biosynthetic pathway in the absence of complex nutrients. TD01-*pmHasA* reached a lower final density *OD*_600 nm_ (0.95) compared to the WT, suggesting that even the most efficient variant competes with primary metabolism for precursors. TD01-*sezHasA* displayed a characteristic prolonged lag phase but continued to climb steadily, eventually approaching the WT density by the 26h mark. TD01-*spHasA* showed the poorest performance, plateauing at an *OD*_600 nm_ of only 0.7.

A significant growth difference was observed between the wild-type and the engineered strains in 60-MMG, as compared to the rich 60-LBG medium, indicating that HA production is bioenergetically expensive. In a defined mineral medium, which was optimized mainly for PHB production in the *H. bluephagenesis* TD01 strain [16,17] the genetically modified cells must divert limited resources from primary metabolic processes to synthesize the nucleotide sugar precursors (UDP-GlcUA and UDP-GlcNAc) required for HA polymerization. These results suggest that while the Halomonas chassis is remarkably robust in a harsh environment, the metabolic flux toward the HA pathway necessitates careful balancing to prevent severe growth decline, especially in non-rich media like 60-MMG.

#### 3.2.2. Growth Kinetics in Nutrient-Rich Medium (60-LBG)

To evaluate the impact of heterologous hasA gene expression on cellular fitness under optimal conditions, the growth kinetics of the engineered *H. bluephagenesis* TD01 strains were monitored in 60-LBG medium over 24 h. As illustrated in Figure 3B, the wild-type (TD01-WT) and the strain harboring the *pmHasA* gene displayed nearly identical growth profiles, both achieving a maximum *OD*_600 nm_ of 2.2 to 2.4. This parity suggests that the biosynthesis of HA mediated by the *P. multocida* enzyme is exceptionally well-tolerated by the host chassis when complex nutrients are abundant, indicating a high level of metabolic compatibility. The physiological response of the other variants differed notably. TD01-*sezHasA* exhibited a slightly extended lag phase compared to the WT, potentially due to the initial metabolic adjustment required for enzyme synthesis; however, it eventually recovered to reach a comparable final biomass. In contrast, the TD01-*spHasA* strain expressing the *S. pyogenes* variant showed a significantly suppressed growth rate and a markedly lower final *OD*_600 nm_ (1.3). This substantial reduction in fitness suggests a higher metabolic burden associated with the *spHasA* enzyme variant within the *Halomonas* chassis. These results underscore that enzyme selection is a critical factor in metabolic engineering, as the specific catalytic properties or protein folding requirements of different variants can significantly influence the balance between product yield and host viability.

### 3.3. Comparative Performance of Recombinant HAS Enzymes on HA Yields

A comparative study was conducted to evaluate the biosynthetic performance of three distinct hyaluronan synthase enzymes, pmHAS, sezHAS, and spHAS, heterogeneously expressed in *H. bluephagenesis* TD01. When cultivated in 60-LBG medium, all three recombinant strains successfully synthesized HA but displayed significant variations in biomass accumulation, titer, and polymer chain length (Table 2). The strain harboring the Class II synthase from *P. multocida* (*pmHasA*) significantly outperformed the Class I enzymes from the *Streptococcus* species across all measured parameters. It achieved a peak HA titer of 0.88 g/L. This represents a statistically significant increase (*p* < 0.0001) compared to the *Streptococcus*-derived variants. In contrast, the *Streptococcus*-derived synthases exhibited lower production efficiencies; TD01-*sezHasA* produced HA with a titer of 0.78 g/L. TD01-*spHasA* displayed the poorest overall performance, with the lowest biomass accumulation (cell dry weight; CDW) of 2.57 g/L and the lowest HA titer of 0.518 g/L.

The marked advantage of pmHAS may be attributed to its unique structural architecture. Unlike the Class I enzymes (e.g., sezHAS and spHAS), which are integral membrane proteins of ~418 amino acids, the Class II pmHAS (972 amino acids) features a distinct modular domain organization. This dual-domain structure, containing separate active sites for each sugar monomer, facilitates a more processive elongation mechanism. This structural difference, rather than protein size alone, likely enables the production of higher-molecular-weight HA and provides greater catalytic stability when expressed within the specific metabolic and membrane landscape of the haloalkaliphilic *Halomonas* chassis (Figure 2B). Based on the superior titer and high-molecular-weight profile demonstrated in Table 2, the TD01-*pmHasA* strain was selected as the optimal candidate for subsequent bioprocess optimization.

### 3.4. Structural Characterization of the HA Polymer

To confirm the identity and purity of the biosynthetic products from the recombinant strains (TD01-*pmHasA*, TD01-*sezHasA*, and TD01-*spHasA*), the polymers were subjected to a rigorous purification process involving repeated ethanol precipitation and deproteinization. The resulting HA solutions were filtered through 0.22 µm syringe filters to remove low-molecular-weight impurities and subsequently lyophilized. Structural elucidation of the purified products was performed using Electrospray Ionization Mass Spectrometry (ESI-MS) in negative ionization mode and ^1^H-NMR spectroscopy.

#### 3.4.1. Mass Spectrometric Analysis (ESI-MS)

**Figure 4:**
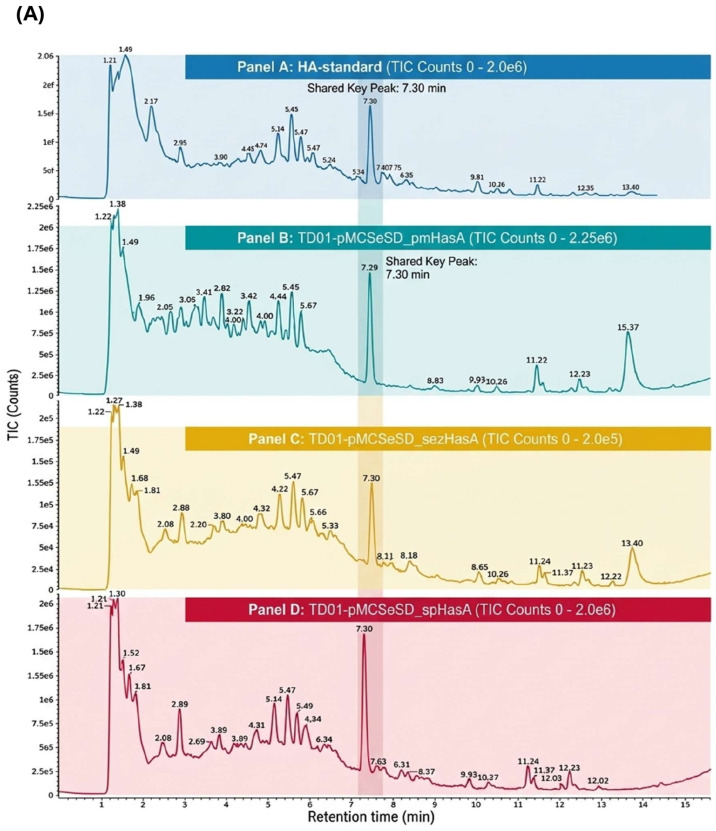

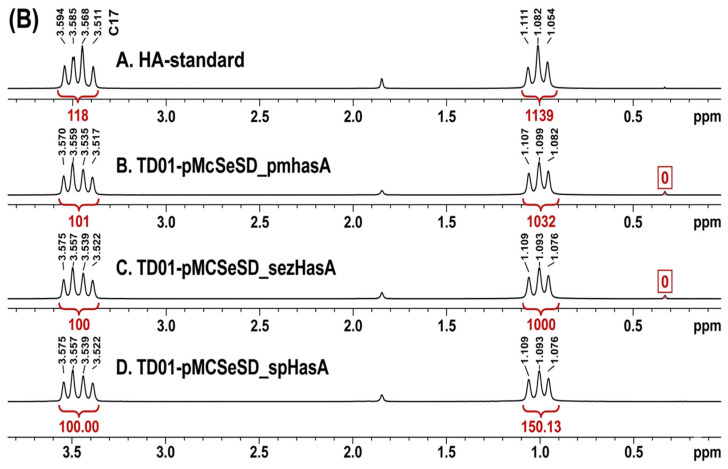
Structural validation of recombinant hyaluronic acid (HA) was confirmed using mass spectrometry (MS) and ^1^H-NMR. (**A**) MS analysis identified characteristic hyaluronan disaccharide repeat fragment ions that matched the HA standard, confirming chemical identity. (**B**) ^1^H-NMR comparison with a commercial standard showed a high degree of overlap and matching diagnostic signals, confirming the correct primary structure of the produced polysaccharide. The area of a signal in an NMR spectrum is directly proportional to the relative number of protons (^1^H atoms) that produce that signal. The red numbers provide a precise way to determine the ratio of different types of protons in a sample.

ESI-MS was employed to characterize the specific functional groups and molecular ions following enzymatic digestion with hyaluronidase. The negative ion mass spectrum (Figure 4A) of the synthesized HA displayed characteristic ion peaks at *m*/*z* 304.91419 and 566.76052. Notably, the experimental samples exhibited a prominent ion at *m*/*z* 379.111476, which aligns with the *m*/*z* 379.31664 observed in the commercial HA standard [43]. This consistency confirms that the primary building blocks of the biopolymer produced by the *Halomonas* chassis are identical to analytically pure hyaluronic acid. Comparative data showed that peaks at 1.2–1.5 min, 2.9 min, 5.4 min, and 7.30 min were common to both the HA standard and all TD01 recombinant strains. This high degree of profile similarity indicates that the recombinant HasA strains produce a profile containing the core HA components.

#### 3.4.2. Proton Nuclear Magnetic Resonance (^1^H-NMR)

The ^1^H-NMR spectra of the synthesized HA samples were compared against a standard reference to validate the presence of characteristic glycosidic linkages and functional groups (Figure 4B). The following chemical shifts (δ) were identified, matching established literature values for bacterially derived HA [44,45]. The N-acetyl group, a distinct signal between 1.054 and 1.07 ppm, corresponds to the methyl protons (CH_3_) of the N-acetyl glucosamine (GlcNAc) residue [46]. Signals observed between 3.50 and 3.60 ppm strongly suggest the presence of ß (1 → 4) and (1 → 3) glycosidic linkages. Specific resonances were detected for protons associated with CH_2_, NH, and OH groups at 3.50, 3.72, and 3.85 ppm, respectively. The methine (CH) group protons were identified in the range of 3.52–3.59 ppm. The alignment of these methylene and acetamido signals in the shielded region of the spectrum confirms the successful biosynthesis of HA with high structural integrity and the correct repetitive disaccharide unit architecture.

### 3.5. Impact of Hyaluronan Synthase Origin on Molecular Weight (M_w_)

The molecular weight of hyaluronic acid (*M_w_*) is an intrinsic property governed by the specific catalytic mechanisms of the hyaluronan synthase (HAS) enzyme. When expressed in the *H. bluephagenesis* TD01 chassis, HAS enzymes sourced from different species of *S. equi* subsp. *zooepidemicus*, *S. pyogenes*, and *P. multocida* produced diverse *M_w_* profiles under identical shake-flask cultivation conditions. This variability is consistent with established models of HAS kinetics, where the rate of chain elongation versus chain termination is enzyme-specific [47]. We validated the influence of enzyme origin by characterizing the HA produced by the three recombinant strains: TD01-(pMCSeSD-*pmHasA*, pMCSeSD-*sezHasA*, and pMCSeSD-*spHasA*) (Table 2).

Our findings demonstrate that both HA titers and molecular weights differed significantly across the three variants (Supplementary Material Figure S4 and Table S6). The high *M_w_* profile (TD01-pMCSeSD-*pmHasA*) harboring the *P. multocida pmHasA* synthase produced the highest molecular weight HA at 1.15 MDa. This variant also correlated with the highest titer, suggesting superior metabolic integration. For the intermediate *M_w_* profile (TD01-*spHasA*), the *S. pyogenes* variant yielded an intermediate molecular weight of 1.05 MDa. For the lower *M_w_* Profile (TD01-pMCSeSD-*sezHasA*), despite being a common industrial source, the *S. zooepidemicus* variant produced the lowest-molecular-weight HA in this host, measured at 0.99 MDa. These variations in *M_w_* underscore the importance of pmHAS (Class II), which often outperforms Class I enzymes like spHAS or sezHAS in terms of polymer length, not its total mass, but its domain architecture and mechanism of translocation. Class I (e.g., sezHAS): These are integral membrane proteins. They have to simultaneously catalyze the reaction and translocate the growing HA chain through the plasma membrane. This pore-forming requirement can limit the chain length if the enzyme–membrane interaction is disrupted. Class II (pmHAS) is a soluble, “dual-domain” enzyme (though it associates with the membrane). It has two distinct active sites, one for each sugar. Its mechanism for holding and extending the chain is structurally different and generally more “processive,” meaning it hangs onto the polymer for a longer time before letting go, resulting in a higher molecular weight (HMW-HA).

### 3.6. Optimization of Induction for HasA Gene Expression

To evaluate the feasibility of the Next-Generation Industrial Biotechnology (NGIB) platform in a controlled environment, the performance of the engineered strains was examined across a gradient of L-arabinose concentrations (0.025% to 1% *w*/*v*). This titration aimed to identify the optimal induction level that balances the metabolic flux between biomass accumulation and HA biosynthesis in both 60-LBG and 60-MMG media.

**Figure 5:**
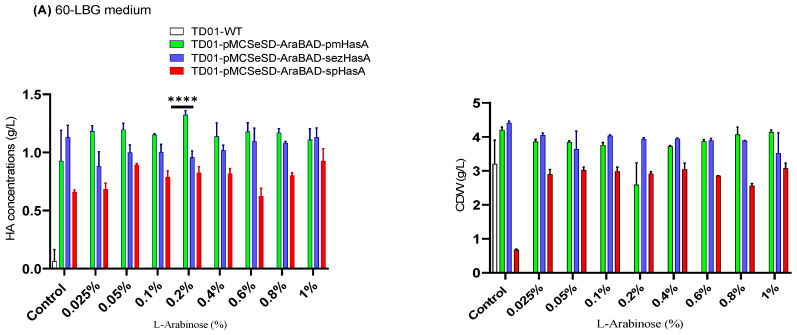

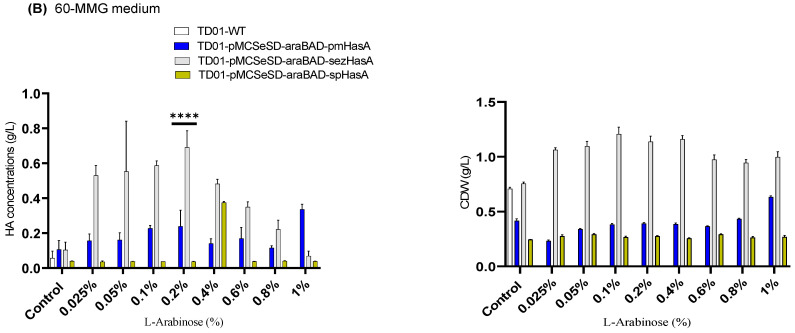
Comparative evaluation of L-arabinose induction on HA production and biomass in recombinant *H. bluephagenesis* TD01. (**A**) performance in rich medium (60-LBG): the left panel displays hyaluronic acid (HA) concentrations (g/L), where bars represent the TD01-pMCSeSD-*araBAD-pmHasA*, TD01-pMCSeSD-*araBAD*-*sezHasA*, and TD01-pMCSeSD-*araBAD*-*spHasA* strains, respectively. The right panel illustrates the corresponding cell dry weight (CDW, g/L), highlighting the differential metabolic impact of each HasA variant on host biomass after 72 h of cultivation. (**B**) performance in mineral medium (60-MMG): The left panel illustrates HA concentrations (g/L), representing the *pmHasA*, *sezHasA*, and *spHasA* strains, respectively. The right panel shows the corresponding CDW (g/L), highlighting the significant growth advantage of the TD01-*sezHasA* strain in the mineral environment relative to the *pmHasA* and *spHasA* variants. Induction was performed across a gradient of L-arabinose concentrations (0.025% to 1%) for 72 h. All experiments were conducted in triplicate; error bars denote the standard deviation from the mean. The wild-type strain (TD01-WT) served as the negative control for HA synthesis across all conditions. The asterisk (****) denotes a statistically significant (*p* < 0.0001) peak in HA production for the *pmHasA* and *sezHasA* strains at an 0.2% L-arabinose induction level.

As shown in Figure 5A, all three recombinant strains successfully synthesized HA across the inducer range, while the wild-type (WT) control exhibited no detectable HA production. TD01-pMCSeSD-*araBAD-pmHasA* emerged as the superior variant in the rich medium (60-LBG), reaching a peak HA titer of 1.3 g/L at a concentration of 0.2% L-arabinose. Increasing the inducer concentration beyond 0.2% did not yield further improvements. This indicates possible pathway saturation or metabolic limitation where elevated protein expression begins to impose a deleterious stress response on the host. The cell dry weight (CDW) for WT, TD01-pMCSeSD-*araBAD*-*pmHasA*, and TD01-pMCSeSD-*araBAD*-*sezHasA* remained stable between 3.5 and 4.5 g/L. Notably, the TD01-pMCSeSD-*araBAD*-*spHasA* strain, which previously showed growth defects, exhibited a slight biomass “recovery” as the inducer concentration increased, though it remained consistently lower than the other variants. The *p*-value (*p* < 0.0001) is strongly significant, and the *pmHasA* strain is the superior producer. R^2^ = 0.96 and 0.98. A lack-of-fit *p*-value > 0.05 indicates the model was adequate. A Tukey’s post hoc test revealed that the *pmHasA* strain at 0.2% L-Arabinose produced a significantly higher HA concentration (1.32 ± 0.03 g/L) compared to all other strains (*p* < 0.01).

The metabolic behavior in the mineral medium (60-MMG) diverged significantly from the results observed in the rich medium (Figure 5B), highlighting the impact of nutrient availability on enzyme performance. Interestingly, while *pmHasA* dominated in rich media (60-LBG), TD01-*sezHasA* was the most robust producer in the mineral environment. It achieved a peak HA concentration of 0.69 g/L at 0.2% L-arabinose. This superior yield was directly correlated with biomass retention; the *sezHasA* strain demonstrated the highest CDW (1.2 g/L) in this nutrient-limited environment compared to the other tested strains. In contrast, both the *pmHasA* and *spHasA* strains struggled to accumulate significant biomass (<0.5 g/L) in 60-MMG, leading to reduced HA titers. These findings suggest that the sezHAS variant may be better adapted to the metabolic constraints of growth in mineral-based seawater media, whereas *pmHasA* requires the supplementary precursors or energy provided by complex organic nitrogen sources to reach its full catalytic potential. Furthermore, 0.2% L-arabinose was identified as the best concentration for maximizing induction efficiency while maintaining cellular fitness across both media types. In this specific medium (60-MMG), the *sezHasA* strain performs significantly differently than the others, often showing higher yields at moderate induction. The model has a *p*-value < 0.0001 and correlation (R^2^) = 0.95–0.97. All points fall well within a 20% margin, meaning the model accurately represents the experimental space. Post hoc analysis showed that the TD01-*sezHasA* strain reached a significantly higher peak titer at 0.2% induction (0.69 ± 0.09 g/L) compared to the other strains under the same conditions.

The araBAD system is prized because it is both tightly repressed and dose-dependent (the amount of inducer equals the amount of protein). In the absence of L-arabinose, the AraC protein binds to the O_2_ and *I*_1_ operators, causing the DNA to loop. This physical loop blocks RNA polymerase from accessing the P*_BAD_* promoter, ensuring almost zero expression of the HA synthase (HAS) or precursor genes. In the presence of L-arabinose, Arabinose binds to AraC, changing its conformation. The DNA loop breaks, and AraC instead helps to recruit RNA polymerase to the promoter. Growth–production decoupling using *P_BAD_* allows the *Halomonas* population to grow to a high density first. Once the cell is built (high biomass), adding arabinose switches the focus to product manufacturing (HA synthesis). This separation prevents the HA production from declining the growth rate. Also, the araBAD system allows for a moderated rate of protein synthesis. This ensures that the HAS enzymes are folded and inserted into the membrane correctly without triggering the cell’s envelope-stress responses, which would otherwise degrade the enzymes and lower yield.

### 3.7. Optimization of HA Production Medium Components Using One-Factor-at-a-Time (OFAT)

In our preliminary optimization of the induction systems (Section 3.5 and Section 3.6), the strain *H. bluephagenesis* TD01-pMCSeSD-*araBAD-pmHasA* consistently outperformed other candidates in terms of both total hyaluronic acid (HA) titer and high-molecular-weight (HA-Mw) production. Therefore, the metabolic response of the *H. bluephagenesis* TD01-pMCSeSD-*araBAD-pmHasA* strain was studied under various culture conditions to maximize production efficiency.

#### 3.7.1. Effect of Nitrogen Sources, NaCl Concentrations, and Initial pH on HA Production

##### The Effect of Nitrogen Sources on HA Production

**Figure 6:**
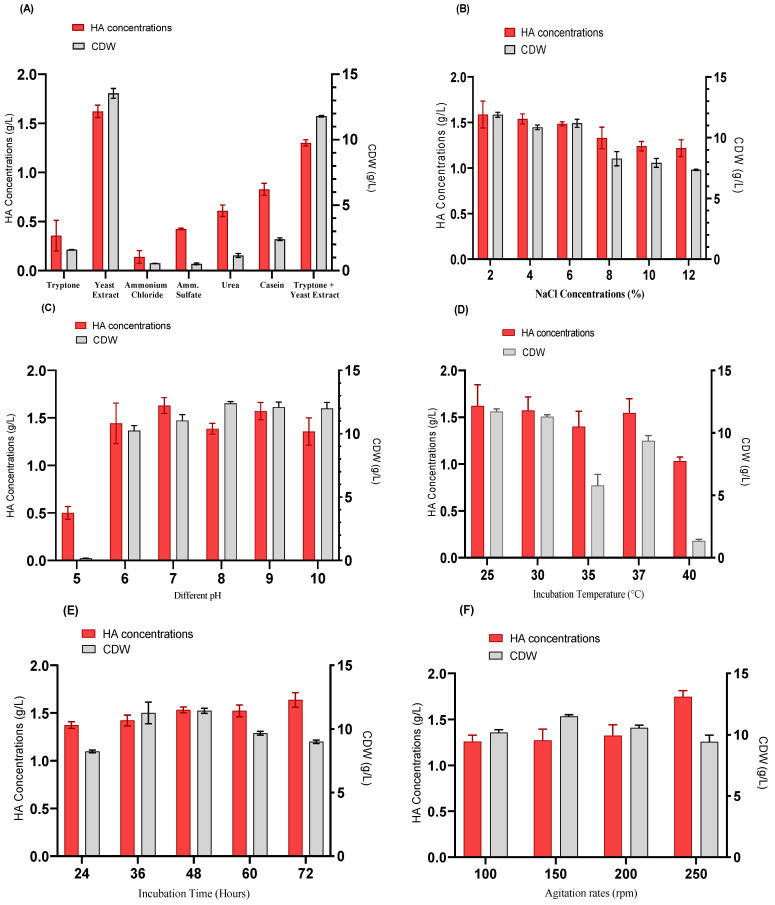
Optimization of HA production medium components using one-factor-at-a-time (OFAT) using *H. bluephagenesis* TD01-pMCSeSD-*araBAD-pmHasA*. (**A**) Effect of nitrogen sources (organic and inorganic nitrogen sources): the comparative effects of seven different nitrogen sources. (**B**) Effect of different NaCl concentrations on the engineered strain’s performance under NaCl concentrations ranging from 2% to 12%, at 72 h of cultivation, where a significant decrease relative to the 2% is noted starting at 8% NaCl. (**C**) Effect of initial culture pH across a pH range from 5 to 10. HA concentrations (g/L) peaked at pH 7, and CDW (g/L) peaked at pH 8. (**D**) Effect of incubation temperature for incubation temperatures ranging from 25 °C to 40 °C, where the HA concentrations were stable up to 37 °C and a significant decline at 40 °C, and CDW indicated optimal growth at lower temperatures (25–30 °C) and severe growth inhibition at 40 °C. (**E**) Effect of incubation time profile, at 24–72 h, showing the steady increase in HA concentrations over time, with significant gains noted after 48 and 72 h relative to the 24 h baseline; the corresponding CDW peaks at 48 h before declining significantly at 60 and 72 h. (**F**) Effect of agitation rates (influence of dissolved oxygen levels, controlled by stirring speeds). The impact of varying the stirring speed (100, 150, 200, and 250 rpm) on the HA fermentation efficiency is shown; the HA concentrations show a significant peak at 250 rpm while the corresponding CDW in moderate agitation (150 rpm) was optimal for growth. All charts display the HA concentrations (g/L) and corresponding CDW (g/L). Data are presented as the mean of triplicate experiments with error bars representing the standard deviation.

As illustrated in Figure 6A, the selection of the nitrogen source exerted a profound impact on both HA concentration and CDW. Organic nitrogen sources, particularly yeast extract, proved the most effective, yielding a peak HA concentration of 1.6 g/L and a maximum CDW was 13.5 g/L. While the combination of tryptone and yeast extract also supported robust performance, it resulted in a slightly lower HA yield (1.3 g/L) compared to yeast extract alone. In contrast, inorganic nitrogen sources such as ammonium chloride and ammonium sulfate, as well as urea and casein, resulted in significantly lower biomass and HA yields (<0.5 g/L). These results demonstrate that while *H. bluephagenesis* TD01 is a versatile chassis; the provision of complex organic nitrogen is critical for supporting the high metabolic demands of both rapid cell growth and heterologous polysaccharide synthesis. Notably, the supplementation of yeast extract more than tripled the biomass compared to other single organic sources, identifying it as an essential component for potential industrial scale-up. For HA and cell biomass values, the *p*-value (<0.0001) was extremely significant, R^2^ (coefficient determination) was 98.14% and 99.80%, and the adjusted R^2^ was 97.74% and 99.71%, respectively, which confirms the model’s strength. The lack of fit was not applicable, but the R^2^ indicates the model was highly adequate and precise.

##### The Effect of NaCl Concentrations on HA Production

To evaluate the halotolerance and metabolic robustness of the engineered platform, the performance of the TD01-pMCSeSD-*araBAD-pmHasA* strain was monitored across a range of NaCl concentrations (2% to 12%). As illustrated in Figure 6B, the strain demonstrated remarkable stability at lower-to-moderate salinities. Peak performance was observed at 2% NaCl, yielding an HA concentration of 1.6 g/L, and the CDW was 12 g/L. While both HA titers and biomass remained statistically comparable between 2% and 6% NaCl (*p* > 0.05), a significant physiological shift occurred as salinity increased further. At 8% NaCl, the biomass dropped sharply to 8 g/L (*p* < 0.0001), likely due to the increased energetic cost of maintaining osmotic balance. Notably, despite this decline in growth, the strain remained metabolically active even under extreme salinity (12% NaCl), still producing over 1.0 g/L of HA. This capability underscores a unique advantage of the *Halomonas* chassis and its ability to maintain heterologous HA synthesis under hypersaline conditions that would typically inhibit traditional industrial hosts. This metabolic resilience confirms that *H. bluephagenesis* TD01-pMCSeSD-*araBAD-pmHasA* is a potent candidate for HA production in non-sterile, open fermentation systems, where high salinity can be leveraged to prevent microbial contamination. For HA and cell biomass values, the *p*-values (0.0017 and <0.0001) were statistically significantly different. For R^2^, the coefficient of determination is 76.6% and 96.47%, and the adjusted R^2^ is 67% and 95%, respectively. The lack of fit (*p*-value) = 0.142 and 0.114, and more than >0.05 was good and highly precise.

##### The Effect of Initial pH on HA Production

Given that *H. bluephagenesis* TD01 is naturally adapted to alkaline environments, the optimal pH range for HA biosynthesis and cellular growth was investigated in the TD01-pMCSeSD-*araBAD-pmHasA* strain. As shown in Figure 6C, the strain exhibited a broad tolerance to pH levels ranging from neutral to highly alkaline but performed poorly under highly acidic conditions. At pH 5, growth was nearly completely inhibited, resulting in negligible biomass and low HA titers (0.5 g/L). Optimal HA production was achieved at pH 7, reaching 1.6 g/L. Interestingly, while HA concentrations remained relatively stable and high across the pH 6–10 range, CDW continued to increase as the medium became more alkaline, peaking at pH 8 with a value of 12.5 g/L. For the HA and cell biomass values, the *p*-value (0.0001) is highly significant, R^2^ (coefficient determination) is 93.52%, 99.53%, and the adjusted R^2^ = 90.81% and 99.43%, respectively. The lack of fit (*p*-value) = 0.018 was good. The optimal peak for HA values was located between pH 7 and 9, and the maximum response was near pH 7.5 to 8.0. The contour map for biomass suggests an operating range between 8.0 and 9.5.

The ability of the strain to maintain high levels of HA production even at pH 10 (>1.3 g/L) further validates its suitability for open, non-sterile fermentation. Such alkaline conditions, combined with the previously noted halotolerance, provide a robust defense mechanism by naturally inhibiting the growth of common microbial contaminants. These findings reinforce the strength of the NGIB platform, demonstrating that TD01-pMCSeSD-*araBAD-pmHasA* not only tolerates hypersaline environments but thrives at high pH, making it an ideal candidate for low-cost, open-tank industrial production.

#### 3.7.2. Effect of Temperature, Incubation Time, and Agitation Speed on HA Production

##### The Effect of Incubation Temperature

Temperature is a critical factor in industrial fermentation, influencing both enzymatic kinetics and cellular membrane integrity. The performance of the TD01-pMCSeSD-*araBAD-pmHasA* strain was evaluated across a temperature range from 25 °C to 40 °C. As shown in Figure 6D, HA production remained remarkably stable across a broad thermal window. No statistically significant differences in HA titers were observed between 25 °C and 37 °C, with concentrations maintaining a steady range of 1.4–1.6 g/L. However, increasing the temperature to 40 °C led to a significant reduction in HA yield to 1.0 g/L (*p* < 0.05). Cellular growth exhibited a more sensitive response to thermal changes than HA synthesis. The CDW was optimal at 25 °C and 30 °C, yielding 11.5 g/L. Interestingly, a dip in biomass was observed at 35 °C, followed by a partial recovery at 37 °C, before a sharp and highly significant decline at 40 °C, where growth was severely limited to 1.3 g/L (*p* < 0.0001). For HA and cell biomass values, the *p*-value (0.0143) is statistically significant and extremely significant, respectively. R2 (coefficient of determination) = 68.23% and 98.22% and adjusted R^2^ = 55.52% and 97.51%, respectively. Lack-of-fit (*p*-value) = 0.218 and 0.003, respectively, which was not significant. Contour plot interpretation explained a clear negative correlation, and the optimization is clearly located at 25 °C. The plateau zone was between 25 and 37 °C.

These results indicate that while the *pmHasA* enzyme is functionally robust up to 37 °C, the ideal operating window for maximizing both biomass and HA titer is between 25 °C and 30 °C. The thermal elasticity of HA production up to 37 °C offers a significant practical advantage for industrial applications. This robustness suggests that the fermentation process is resilient to minor temperature fluctuations, which are common in large-scale industrial bioreactors.

##### The Effect of Fermentation Time

To determine the optimal harvest time for the engineered platform, a 72 h time-course study was conducted using the optimized TD01-pMCSeSD-*araBAD-pmHasA* strain. As shown in Figure 6E, HA production and cellular biomass accumulation followed distinct temporal patterns. The cell dry weight (CDW) increased rapidly during the first 36 h, reaching a plateau of 11.5 g/L between 36 and 48 h. Beyond 48 h, a significant decline in biomass was observed (*p* < 0.001), likely due to nutrient depletion and the onset of the stationary or decline phases. In contrast, HA concentrations continued to rise steadily throughout the entire 72 h period, showing a significant increase from 1.38 g/L at 24 h to a peak of 1.65 g/L at 72 h (*p* < 0.01). This continued accumulation suggests that the HA biosynthetic machinery remains active even as the total viable biomass begins to decrease. These findings indicate that while 48 h is optimal for maximizing biomass, extending the incubation to 72 h provides the highest total HA yield for this batch fermentation process.

From an industrial perspective, the marginal gain of approximately 0.15–0.2 g/L of HA between 48 and 72 h represents a significant increase in total yield. Despite the onset of the cellular decline phase, the continued productivity of the biosynthetic pathway justifies the extended fermentation duration to maximize the efficiency of the batch process. For HA and cell biomass values, the *p*-value (ANOVA) = 0.0013 was highly significant. R^2^ (coefficient of determination) was 0.752 and 0.741, and the adjusted R^2^ was 0.733 and 0.698, respectively. The lack-of-fit (LOF) test for HA concentration resulted in a *p*-value greater than 0.05 and was not significant. For cell biomass values, the lack-of-fit (LOF) test result was less than 0.001, which was significant. For the contour plot interpretation, the highest biomass is observed around 36 h to 48 h, and the mathematical optimum occurs at approximately 47.9 h.

##### The Effect of Agitation Rate

To finalize the physical parameters of the fermentation process, the impact of oxygen transfer driven by the agitation rate on the metabolic partitioning between cell growth and HA biosynthesis was examined. As aerobic fermentation typically requires efficient oxygen transfer for high-titer production, the performance of the TD01-pMCSeSD-*araBAD-pmHasA* strain was evaluated at agitation rates ranging from 100 to 250 rpm.

As shown in Figure 6F, the agitation rate exerted divergent effects on cell growth versus HA synthesis. The CDW peaked at a moderate agitation rate of 150 rpm, reaching 11.5 g/L. However, increasing the agitation to 250 rpm resulted in a significant decrease in biomass to 9.5 g/L (*p* < 0.001), likely due to increased mechanical shear stress on the cells. In contrast, HA production was markedly enhanced at the highest agitation rate. While HA concentrations remained stable at 1.25 g/L between 100 and 150 rpm, a sharp increase to 1.74 g/L was observed at 250 rpm (*p* < 0.01). This suggests that while higher agitation rates may hinder total biomass accumulation, improved dissolved oxygen levels or mechanical stimuli significantly increase the specific productivity of the HA synthase enzyme. Ultimately, although the 250-rpm condition reduced total biomass, the resulting increase in HA titer (1.8 g/L) represents a favorable metabolic trade-off. Consequently, 250 rpm was selected as the optimal agitation speed to maximize overall production efficiency. For HA and cell biomass results, the *p*-value = 0.0008 with a significant effect. R^2^ (coefficient of determination) was 0.834 (83.4%) and 0.8036 (80.36%) with observed variation in the values, respectively. The adjusted R^2^ was 0.797 and 0.7599. The lack of fit (LOF) was 0.219 and 0.0392 (with non-significant (*p* > 0.05) and significant values, respectively). The contours show a rising ridge at the higher rpm values (250 rpm), indicating that HA concentration and cell biomass are very sensitive to agitation in that region. The optimum agitation rate is likely above 250 and 150 rpm, respectively. The sharp increase at 250 rpm suggests that higher agitation likely overcomes oxygen or nutrient mass transfer limitations, leading to a significant boost in HA production. While the HA concentration was still rising at 250 rpm, the cell biomass was already well past its prime at that speed. To maximize both, we will need to find the rate at which these two conflicting trends intersect.

### 3.8. Plackett–Burman Analysis

The Plackett–Burman design (PBD) was employed as a robust statistical screening tool to identify the primary factors significantly affecting HA production. A total of eight variables, along with three dummies variables, were evaluated across 12 experimental runs. Each factor was tested at two levels, high (+1) and low (−1), to determine its relative influence on productivity. The experimental design and the corresponding HA yields are summarized in Table 3.

**Figure 7:**
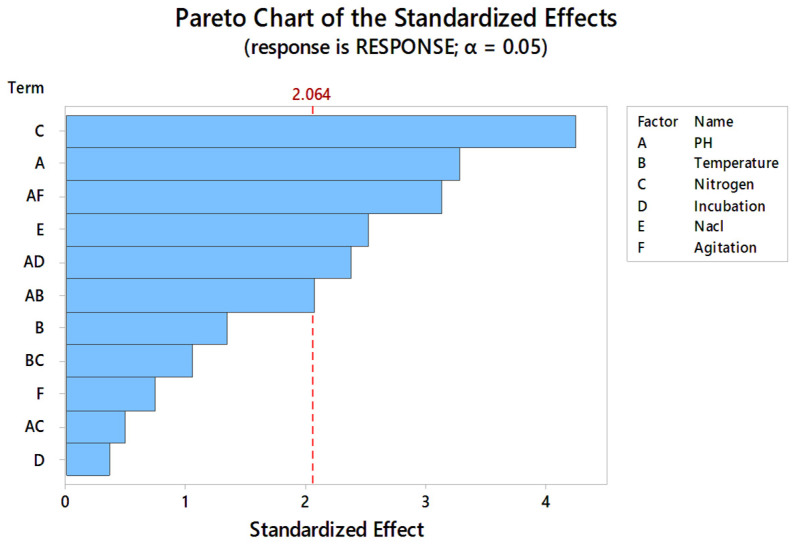
The Pareto chart obtained for the HA optimization by Plackett–Burman analysis. The Red Number (2.064) is the Critical *t*-Value. a level of confidence, specified here as *α* (alpha) = 0.05. This number is the “cutoff” from the *t*-distribution for a 95% confidence level (100%–5%). The height of each bar represents how strong that factor’s effect is, scaled by its measurement error. Any term whose bar crosses this line is statistically significant. Terms whose bars are shorter than the line are considered “not statistically significant” at the 95% level.

Analysis of Variance (ANOVA) was performed to validate the model’s reliability (Table 4). The resulting model F-value of 16.85 indicates that the model is highly significant, with only a 0.01% probability that such a value could occur due to experimental noise. Model terms with *p*-values < 0.0500 were considered statistically significant. Among the factors tested, initial pH (A, *p* = 0.003), nitrogen sources (C, *p* < 0.001), and NaCl concentration (E, *p* = 0.018) were found to have significant effects on HA production. In contrast, temperature (B, *p* = 0.190), incubation time (D, *p* = 0.722), and agitation rate (F, *p* = 0.463) did not reach statistical significance as individual model terms. However, evaluation via the Pareto chart (Figure 7) provided further nuance; while incubation time and agitation rate showed high *p*-values, the Pareto analysis highlighted their relative importance in the overall process hierarchy when compared to the dummy variables. Based on the combined results of the *p*-values and the Pareto chart, three pivotal factors, initial pH, nitrogen sources, and NaCl concentration, were selected for further optimization, where a pH of 9, temperature of 25 °C, yeast extract concentration of 15 g/L, 6% NaCl, and agitation rate of 200 rpm were the best conditions for a HA yield of 2.12 g/L. The optimal concentrations and the interactions between these three variables were subsequently evaluated using a central composite design (CCD).

### 3.9. Statistical Optimization of HA Production by Response Surface Methodology (RSM)

Statistical optimization of the primary factors influencing HA production and the complex interactions between variables was conducted using Response Surface Methodology (RSM). Based on the previous Plackett–Burman analysis and Pareto chart, three factors, initial pH (A), NaCl concentration (B), and nitrogen source (C), were identified as having significant effects. A central composite design (CCD) was subsequently employed to evaluate these critical factors across five levels (–1.68, –1, 0, +1, and +1.68). A total of 20 experimental runs were performed, as summarized in Table 5. The experimental yields of HA ranged from 1.15 g/L to 2.38 g/L. The maximum concentration (2.38 g/L) was achieved in Run 15, corresponding to an axial level of the nitrogen source (+1.68) at pH 8.0 and 4% NaCl. This result aligned closely with the model’s predicted response, demonstrating high precision. Conversely, the lowest yield (1.15 g/L) occurred at pH 9.0 with 6% salinity and a lower nitrogen level (–1). This suggests that while *H. bluephagenesis* TD01 is haloalkaliphilic, the combined stress of excessive salinity and nitrogen limitation hinders HA synthesis.

The statistical significance of the quadratic model was confirmed via Analysis of Variance (ANOVA) (Table 6). The model F-value of 16.85 and a low *p*-value (*p* < 0.0001) indicate that the model is highly significant, with only a 0.01% chance that the results were due to noise. Among the linear terms, nitrogen source (C) was the most significant (*p* = 0.0186). However, the model revealed critical quadratic effects for all three variables (A^2^, B^2^, and C^2^), suggesting that HA production follows a curved trajectory with distinct optimal peaks for pH, salinity, and nitrogen levels. Notably, the interaction between NaCl and the nitrogen source (BC) was statistically significant (*p* = 0.0485), implying that the effect of salinity on HA yield is dependent on the nitrogen source provided. The non-significant lack of fit (*p* = 0.2209) further confirms that the model effectively represents the experimental data. The model’s reliability was supported by a high coefficient of determination (R^2^ = 0.9381), explaining 93.81% of the variability in HA production. A low coefficient of variation (CV = 6.92%) and an Adeq precision ratio of 14.229 (well above the threshold of 4) demonstrate a strong signal-to-noise ratio and high experimental precision. To provide a quantitative relationship between the independent variables and the HA yield, the following second-order polynomial equation was derived:

Yield (HA) = −4.96142 + 1.48613A + 49.07868B + 0.407871C − 4.42508AB − 0.028914AC + 4.99587BC − 0.080796A^2^ − 189.37181B^2^ − 0.111973C^2^ (2)

**Figure 8:**
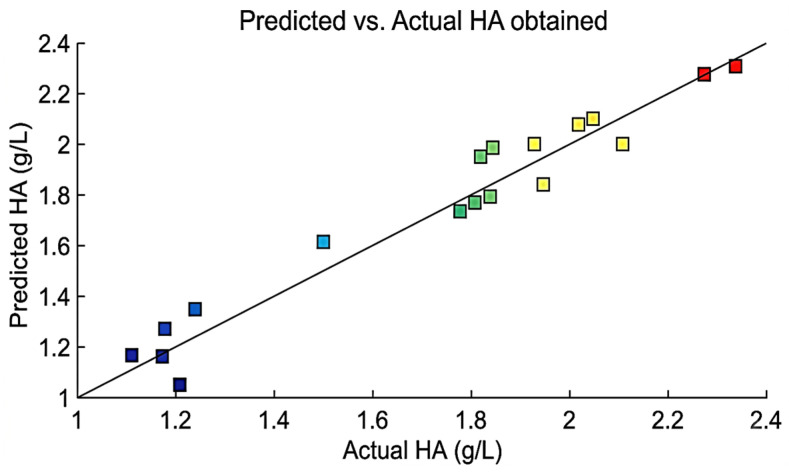
The linear correlation plot of the predicted vs. actual HA concentration (g/L), demonstrating the model accuracy. All points are tucked quite closely along the 1:1 line. The dashed lines represent a ± 20% deviation from the actual values.

**Figure 9:**
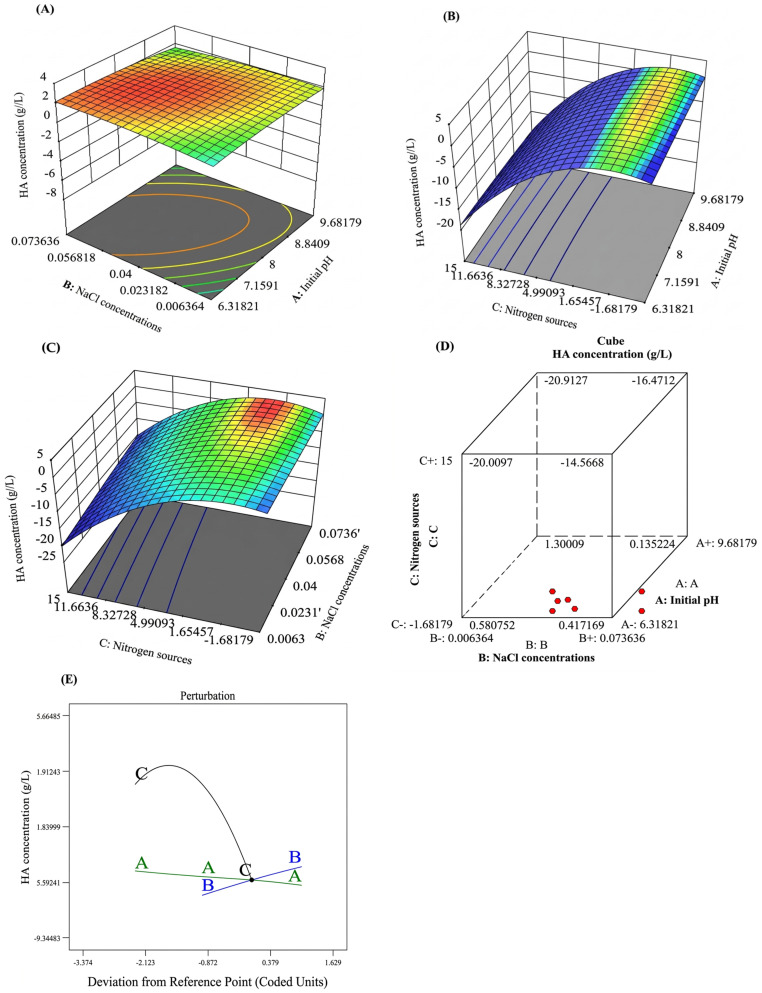
Response surface analysis and interaction profiles for HA production optimization. (**A**–**E**) 3D response surface and contour plots panels illustrate the interactive effects of the primary independent variables on hyaluronic acid (HA) yield (g/L). The color gradient from (blue to red or orange) represents a significant increase in HA concentration (R1), ranging from 1.13 to 2.35 g/L. (**A**) Interaction of initial pH and NaCl concentration, demonstrating the optimal ridge for HA synthesis at near-neutral to slightly alkaline conditions across moderate salinities. (**B**) Interaction of initial pH and nitrogen source, visualized at a constant NaCl concentration of 2%. The steep response along the C-axis underscores the vital importance of nitrogen quality in maximizing HA titers. (**C**) Interaction of NaCl concentration and nitrogen source, visualized at a constant pH of 7.0. The elliptical nature of the contours indicates a strong synergistic effect between salinity and nitrogen availability. (**D**) Cube plot and (**E**) perturbation analysis. The cube plot maps the predicted HA yield (g/L) at the extreme boundaries of the experimental design space for Factors A = pH, B = NaCl concentrations, and C = nitrogen sources. The perturbation plot illustrates the sensitivity of HA production to changes in each factor while holding others constant at the central point. The stark drop in predicted values at the extreme vertices emphasizes the metabolic sensitivity of *H. bluephagenesis* TD01-pMCSeSD-*araBAD*-*pmHasA* and the necessity of maintaining centralized, optimized parameters to prevent biological suppression. The colors and red dots show the natural variation, or experimental error, inherent in measuring the response (HA concentration), and seeing the spread of the red dots gives a visual estimate of the data’s repeatability. Their location, clustered near the origin of the plot where factors A, B, and C are all at their low ‘-‘ levels (representing the lowest concentrations and pH), identifies them as a set of replicated baseline runs or “center points” for the entire design space. These runs are essential for calculating pure experimental error and testing for curvature in the response.

The relationship between the experimental data and the statistical model was visually assessed using a predicted vs. actual plot (Figure 8), which showed a high degree of correlation along the 45° diagnostic line. 3D response surface plots were generated to further elucidate the interactive effects. The interaction between initial pH and NaCl revealed a distinct “dome” shape, indicating that HA production is maximized at a near-neutral to slightly alkaline pH (8.0 to 9.0) and moderate NaCl concentrations (2% to 4%) (Figure 9A). The elliptical contour lines suggest a significant interaction where the bacterium’s salinity tolerance is modulated by the surrounding pH. The role of nitrogen availability was even more pronounced. The interaction between pH and nitrogen showed a steep incline in HA production as nitrogen levels moved toward yeast extraction (+1), indicating that the magnitude of production is strictly limited by the nitrogen source type (Figure 9B). Furthermore, the NaCl andnitrogen interaction showed that HA yield is maximized at lower salinity levels when supplemented with high nitrogen availability (Figure 9C). This suggests that complex nitrogen sources like yeast extract likely provide essential precursors or osmo-protectants that mitigate the metabolic burden of HA synthesis under hypersaline conditions. Finally, the cube plot and perturbation highlighted that extreme boundary conditions, high salinity, and extreme pH are highly detrimental, leading to metabolic inhibition (Figure 9D,E). This confirms that the ideal fermentation environment requires the carefully balanced, centralized conditions identified by the response surface models.

### 3.10. Effect of L-Arabinose Concentrations on HA Molecular Weight (M_w_)

**Figure 10:**
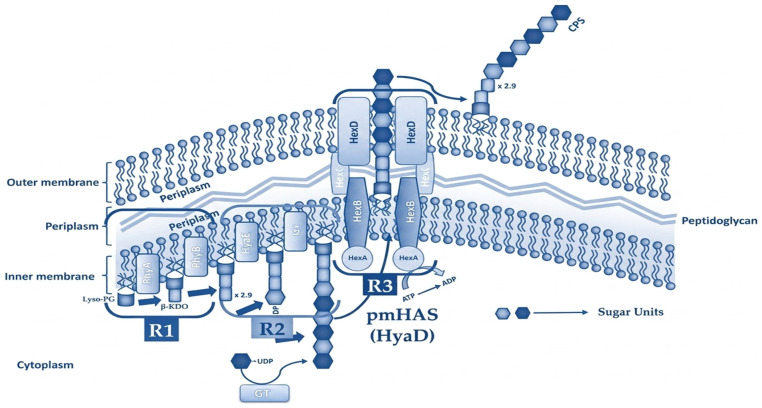
The HA molecular weight controlling in the engineering of *H. bluephagenesis* TD01 harboring pmHAS for HA production (adapted based on [55,56,57]). The classical model of the cell envelope structure type-2 HA biosynthesis, with its crucial components labeled. The pmHAS (HyaD) is the *P. multocida* hyaluronan synthase and its ABC-transporter-dependent biosynthesis pathway. HA is synthesized as a glycolipid on the cytosolic side of the inner membrane and then exported across the cell envelope [58,59]. R1: Contains phyAB, lipAB genes; responsible for lipidation and surface attachment of HA. R2: Contains PmHAS gene; hyaluronic acid polymerization. R3: Contains conserved hexA-D genes, encoding components of the ABC transport system.

To evaluate the impact of the inducible pBAD promoter system on the performance of the engineered *H. bluephagenesis* TD01-pMCSeSD-*araBAD-pmHasA* strain, the production of hyaluronic acid (HA) and its resulting molecular weight (*M_w_*) were monitored across a range of L-arabinose concentrations (0% to 1% *w*/*v*). The results, summarized in Table 7) (Supplementary Material Table S7 and Figure S5), indicate that while CDW and HA concentration remained relatively stable across the tested range, the *M_w_* of the produced HA was highly sensitive to the concentration of the inducer. As shown in the experimental data, the maximum HA yield (1.45 g/L) and a high CDW (9.52 g/L) were achieved at an L-arabinose concentration of 0.6%. Notably, a baseline HA concentration of 1.34 g/L was observed even in the absence of the inducer (0%), with an *M_w_* of 2.4 MDa. This suggests a high baseline activity of the *pmHasA* enzyme within the *Halomonas* chassis (Figure 10), which may be because of the ionic environment and cations that improve HA stability [48,49,50,51,52,53,54].

Interestingly, the data revealed a distinct “dip” in molecular weight at 0.2% L-arabinose (1.5 MDa) before the values climbed sharply again. This phenomenon may suggest a specific metabolic shift or an altered precursor ratio (GlcNAc/GlcA) relative to enzyme concentration at that induction level, temporarily favoring chain termination over elongation. These findings demonstrate that the pBAD system allows for precise control over the physical properties of the HA polymer without compromising overall strain productivity. Tuning molecular weight through inducer gradients, despite the stability of the total yield, increasing the L-arabinose concentration led to a dramatic, non-linear increase in the average molecular weight. The highest *M_w_* was 9.67 MDa at 1% L-arabinose, representing nearly a four-fold increase compared to the non-induced state. Statistical analysis confirmed that these variations were significant (*p* < 0.05), establishing L-arabinose concentration as a critical lever for tuning polymer chain length in *H. bluephagenesis* TD01-pMCSeSD-*araBAD-pmHasA*.

## 4. Discussion

The construction of biosynthetic pathways for hyaluronic acid (HA) in genetically engineered microbes represents a sustainable and safe alternative for producing high-value glycosaminoglycans. In developing an efficient HA-producing cell factory, selecting an appropriate HA synthase (HAS) is paramount as productivity and *M_w_* are largely determined by enzymatic activity [60,61]. While most HA synthases are derived from vertebrates, bacterial variants are primarily found in *Streptococcus* (Group A) and *P. multocida* [62].

In this study, *H. bluephagenesis* TD01-pMCSeSD-*pmHasA* without induction and TD01-pMCSeSD-*araBAD-pmHasA* with induction by L-arabinose exhibited superior HA synthesis activity in *H. bluephagenesis* TD01, resulting in higher HA accumulation (0.88–1.3 g/L; 1.15–2.43 MDa) compared to synthases from other species (Table 2 and Section 3.6). Structural differences, particularly in the transmembrane regions, suggest that *pmHasA*, which lacks the four transmembrane helices found in *Streptococcal* HasA, is better suited to the *H. bluephagenesis* TD01 cytoplasmic membrane microenvironment [63]. These findings align with Gomes et al. [64], who reported that *P. multocida* synthase yielded maximum HA levels of 1.89 g/L. Interestingly, while Wang et al. [53] found *spHasA* to be superior in *C. glutamicum*, our results highlight a medium-dependent performance. Specifically, *sezHasA* performed better in mineral medium (60-MMG), whereas *pmHasA* dominated in rich medium (60-LBG) (Figure 3), suggesting that optimal strain selection is intrinsically linked to the fermentation environment.

The widening gap in performance between wild-type and engineered strains, particularly in mineral media, underscores the high metabolic demand of HA synthesis. This “metabolic burden” often leads to reduced growth rates, a phenomenon previously observed in recombinant *E. coli* and *S. thermophilus* [65]. To confirm the integrity of the product, lyophilized HA samples were characterized using MS and ^1^H-NMR (Figure 4). The detection of peaks at *m*/*z* 304.91 and 566.76 consistent with HA standards (*m*/*z* 379.31), along with chemical shift values similar to those reported by Welti et al. [66], confirmed that the fermentation product was indeed hyaluronic acid.

Optimization of nitrogen sources revealed that yeast extract is essential for industrial scale-up, yielding 1.62 g/L of HA (Figure 6A). Yeast extract serves as a critical nutrient, likely increasing uridine triphosphate (UTP) synthesis via pyrimidine metabolism [67]. Furthermore, the halophilic nature of the host allowed for successful production at 2% NaCl, with the strain maintaining productivity even at 12% NaCl (1.0 g/L) (Figure 6B). This salinity tolerance, combined with a thriving growth profile at pH 8–10, provides a robust defense mechanism against microbial contamination in open-tank fermentation—a core advantage of the NGIB platform.

Physical parameters also played a significant role. Optimal HA levels were achieved at 25–30 °C, although production remained stable at 37 °C (Figure 6D). While biomass peaked at 48 h, extending the fermentation to 72 h maximized HA yield (1.65 g/L), justifying the additional incubation time. Furthermore, an agitation speed of 250 rpm was identified as ideal, likely due to enhanced oxygen transfer and the efficient release of HA capsules from the cell surface into the medium (Figure 6F).

Following the OFAT studies, Plackett–Burman design (PBD) and Response Surface Methodology (RSM) were used to refine the process. The PBD identified the most critical factors, and subsequent central composite design (CCD) optimization led to a 265% increase in HA production. The model predicted a maximum yield of 2.38 g/L at an initial pH of 8.0, 4% NaCl, and elevated yeast extraction levels, which was experimentally validated. The significant interaction between NaCl and nitrogen (B + C) suggests that *Halomonas* requires specific nitrogen availability to mitigate osmotic stress during high-titer polymer synthesis.

The *M_w_* of HA is a critical factor for clinical applications. By modulating the pBAD promoter with L-arabinose, we achieved precise control over polymer elongation. As L-arabinose concentration increased from 0.05% to 1.0%, the *M_w_* shifted from 2.04 MDa to 9.67 MDa (Table 7). A notable “dip” in *M_w_* was observed at 0.2% L-arabinose (1.54 MDa), potentially indicating a metabolic shift in the ratio of precursors (GlcNAc/GlcA) to enzyme concentration that favors chain termination. Also, the relationship between HA yield and HA-*M_w_* in microbial fermentation is typically complex, often presenting as a trade-off where maximizing one makes it harder to achieve the other [67,68]. In spite of that, the superior performance of *pmHasA* in this chassis may be attributed to its unique structure. At 972 amino acids, significantly larger than the 418 amino acids found in other isoforms, *pmHasA* likely possesses a higher capacity for polymer elongation and secretory transport. This confirms that the modulation of transmembrane protein-dependent catalytic synthesis is a viable strategy for tailoring HA properties in haloalkaliphilic hosts (Figure 10).

## 5. Conclusions

This research successfully establishes *H. bluephagenesis* TD01 as a robust and sustainable “Generally Recognized As Safe” (GRAS) microbial platform for the industrial production of hyaluronic acid (HA). By leveraging the principles of Next-Generation Industrial Biotechnology (NGIB), this study demonstrates that the haloalkaliphilic nature of the *Halomonas* chassis allows for high-titer HA biosynthesis under non-sterile, hypersaline, and alkaline conditions, effectively reducing costs associated with energy-intensive sterilization and freshwater consumption.

A comparative analysis of heterologous hyaluronan synthases revealed that the Class II enzyme from *P. multocida* (*pmHasA*) is the superior variant for this host, particularly in nutrient-rich environments. Through systematic statistical optimization using Plackett–Burman design and Response Surface Methodology (RSM), critical factors such as initial pH, nitrogen source (yeast extract), and salinity were identified and refined to achieve a maximum HA yield of 2.38 g/L.

Furthermore, this study provides a powerful method for tailoring the physical properties of HA, demonstrating that the molecular weight can be precisely tuned from 2.04 MDa to 9.67 MDa by modulating L-arabinose induction levels. The structural integrity and purity of the resulting polymers were confirmed through ESI-MS and ^1^H-NMR. Ultimately, these findings offer a scalable, customizable, and economically viable alternative to traditional HA production methods, fulfilling the growing industrial demand for high-quality, safe glycosaminoglycans.

## Figures and Tables

**Table 1:** The plasmids used in this study are listed in Table 1.

Strain/Plasmid	Description	Reference
Strains		
*E. coli DH5a*	F^−^ φ80lacZΔM15 Δ(lacZYA-argF) U169 endA1 recA1 hsdR17 (r_k_^−^, m_k_^+^) supE44 thi−1 gyrA996 relA1 phoA	In this study [32]
*E. coli S17-1 λpir*	TpR SmR recA, thi, pro, hsdR-M+RP4: 2-Tc: Mu: Km Tn7 λpir with integrated conjugal transfer functions (RP4 transfer functions)	In this study
*H. bluephagenesis TD01*	A robust and contamination-resistant microorganism has been developed as a chassis for “Next-Generation Industrial Biotechnology”	In this study
TD01_pMCSeSD-*pmHasA*	*H. sp. TD01* carrying pMCSeSD-*pmHasA*	Developed in this study
TD01_pMCSeSD-*sezHasA*	*H. sp. TD01* carrying pMCSeSD-*sezHasA*	Developed in this study
TD01_pMCSeSD-*spHasA*	*H. sp. TD01* carrying pMCSeSD-*spHasA*	Developed in this study
Plasmids		
pMCSeSD-sfGFP-backbone	Multicopy *E. coli DH5a* and *E. coli S17-1* shuttle vector; *Cm*^R^, replicative	In this study
pMABY-KS-*araC-araBAD* promoter	pMABY-KS carrying *araC-araBAD* promoter gene	In this study
pMCSeSD-*pmHasA*	pMCSeSD carrying *pmHasA* gene	Developed in this study
pMCSeSD-*sezHasA*	pMCSeSD carrying *sezHasA* gene	Developed in this study
pMCSeSD-*spHasA*	pMCSeSD carrying *spHasA* gene	Developed in this study
pMCSeSD-*AraC*-*AraBAD*-*pmHasA*	pMCSeSD-*pmHasA* carrying *AraC-AraBAD* promoter	Developed in this study
pMCSeSD-*AraC-AraBAD*-*sezHasA*	pMCSeSD-*sezHasA* carrying *AraC-AraBAD* promoter	Developed in this study
pMCSeSD-*AraC-AraBAD*-*spHasA*	pMCSeSD-*spHasA* carrying *AraC-AraBAD* promoter	Developed in this study

**Table 2:** HA synthases from different *Streptococcus* species and *P. multocida* recombinant in *H. bluephagenesis*.

Strain	MWHA (Da)	CDW (g/L)	HA Titer (g/L)
*H. bluephagenesis* TD01-pMCSeSD-*pmhasA*	1,151,039 ± 0.1	3.22	0.88
*H. bluephagenesis* TD01-pMCSeSD-*sezhasA*	990,754 ± 0.04	3.11	0.78
*H. bluephagenesis* TD01-pMCSeSD-*sphasA*	1,059,027 ± 0.06	2.57	0.518

HA-Mw values are presented as the mean ± SD. Statistical significance was determined by one-way ANOVA, with each strain compared against *H. bluephagenesis* TD01-WT.

**Table 3:** Plackett–Burman experimental design and yield of HA obtained.

Run Order	Factor 1	Factor 2	Factor 3	Factor 4	Factor 5	Factor 6	HA Conc. (g/L)	Predicted HA Conc. (g/L)
Run ^a^	A: pH	B: Temperature	C: Nitrogen Sources ^b^	D: Incubation Time	E: NaCl Concentrations	F: Agitation Rate	Response (R1) ^c^	Predicted Value
1	7	25	1	72	2%	250	1.82 ± 0.21	1.39
2	7	25	−1	48	2%	200	1.49 ± 0.08	1.57
3	9	37	−1	72	2%	250	1.57 ± 0.09	1.72
4	9	37	1	48	6%	250	2.01 ± 0.18	1.83
5	9	25	1	48	2%	200	1.72 ± 0.08	1.66
6	9	37	−1	72	2%	200	1.73 ± 0.03	1.39
7	9	25	1	72	6%	200	2.12 ± 0.21	1.73
8	7	37	−1	48	6%	200	1.39 ± 0.08	1.66
9	7	37	1	72	6%	200	1.76 ± 0.09	1.8
10	7	37	1	48	2%	250	1.80 ± 0.18	2.12
11	7	25	−1	72	6%	250	1.83 ± 0.08	1.49
12	9	25	−1	48	6%	250	1.66 ± 0.03	1.73

^a^ All runs were 3 replicates. ^b^ 1: yeast extract; −1: tryptone + yeast extract, based on the effect of normal individual factors as a nitrogen source. ^c^ Response (R1); HA concentration values are presented as the mean ± SD.

**Table 4:** The *p*-values obtained from the ANOVA method for the Plackett–Burman analysis.

Source	*p*-Value	Statistical Significance
Model	0.000	Significant
Linear	0.000	Significant
PH	0.003	Significant
Temperature	0.190	
Nitrogen Sources	0.000	Significant
Incubation Time	0.722	
NaCl Concentration	0.018	Significant
Agitation Rate	0.463	

**Table 5:** The actual design of experiments (DoE), the results of the central composite design, and the yield of HA obtained.

Run Order	Factor 1	Factor 2	Factor 3	HA Conc. (g/L) (R1)	Predicted Response
Run ^a^	A: pH	B: NaCl Concentrations	C: Nitrogen Sources ^b^	HA Conc. ^c^ (g/L)	HA Conc. (g/L)
1	7	2%	−1	1.26 ± 0.02	1.22
2	7	2%	1	1.98 ± 0.15	2.05
3	8	7.36%	0	1.83 ± 0.08	1.86
4	9	6%	−1	1.15 ± 0.02	1.17
5	7	6%	−1	1.18 ± 0.03	1.31
6	8	4%	−1.68	1.18 ± 0.04	1.18
7	9.68	4%	0	1.90 ± 0.04	1.90
8	8	0.63%	0	2.02 ± 0.08	1.98
9	6.31	4%	0	1.88 ± 0.07	1.87
10	9	6%	1	2.06 ± 0.01	2.00
11	8	4%	0	1.96 ± 0.07	2.01
12	9	2%	1	2.08 ± 0.01	2.22
13	9	2%	−1	1.52 ± 0.04	1.40
14	8	4%	0	1.93 ± 0.09	2.01
15	8	4%	1.68	2.38 ± 0.05	2.38
16	8	4%	0	2.14 ± 0.05	2.01
17	8	4%	0	2.01 ± 0.06	2.01
18	7	6%	1	2.28 ± 0.09	2.13
19	8	4%	0	2.08 ± 0.04	2.01
20	8	4%	0	1.96 ± 0.01	2.01

^a^ All runs were in 3 replicates. ^b^ 1: yeast extract; 0: tryptone + yeast extract; −1: casein. The weights of nitrogen sources were adjusted as standard preparations based on the effect of normal individual factors as follows: 1 = yeast extract 15 g/L and 1.68 = 25.22 g/L; 0 = tryptone + yeast extract 10 + 5 g/L; −1 = casein 15 g/L and −1.68 = 25.22 g/L. The culture conditions were 25 °C, 200 rpm, and 72 h; 200 rpm, and 72 h; and 200 rpm and 72 h, as obtained and optimized from the Plackett-Burman design results. ^c^ HA conc. (g/L) values are presented as the mean ± SD.

**Table 6:** ANOVA table obtained for the regression model for HA production.

Source	Sum of Squares	df	Mean Square	F-Value	*p*-Value	
Model	2.40	9	0.2668	16.85	<0.0001	Significant
A-A	0.0059	1	0.0059	0.373	0.5546	Not significant
B-B	0.0748	1	0.0748	4.72	0.0548	Significant
C-C	0.1247	1	0.1247	7.88	0.0186	
AB	0.0627	1	0.0627	3.96	0.0747	
AC	0.0067	1	0.0067	0.422	0.5304	Not significant
BC	0.0799	1	0.0799	5.04	0.0485	Significant
A^2^	0.0941	1	0.0941	5.94	0.0350	
B^2^	0.0827	1	0.0827	5.22	0.0454	
C^2^	0.1807	1	0.1807	11.41	0.0070	Significant
Residual	0.1583	10	0.0158			
Lack of Fit	0.1068	5	0.0214	2.08	0.2209	Not significant
Pure Error	0.0515	5	0.0103			
Cor Total	2.56	19				

**Table 7:** Effect of different L-arabinose concentrations on HA yield and molecular weight produced by *H. bluephagenesis* TD01-pMCSeSD-*araBAD-pmHasA*.

L-Arabinose Concentration (%)	HA Concentration (g/L) ^a^	CDW (g/L) ^a^	Average HA Molecular Weight (Da)
0	1.34 ± 0.12	8.54 ± 0.07	2,438,401
0.05	1.07 ± 0.09	9.74 ± 0.57	2,043,548
0.1	1.34 ± 0.12	8.56 ± 0.50	3,564,708
0.2	1.41 ± 0.12	9.26 ± 0.54	1,549,380
0.4	1.27 ± 0.11	10.16 ± 0.60	5,019,643
0.6	1.45 ± 0.13	9.52 ± 0.56	9,212,013
0.8	1.44 ± 0.13	9.78 ± 0.57	7,101,713
1	1.31 ± 0.12	9.13 ± 0.53	9,674,869

^a^ All results are expressed as means ± standard deviation of means. Values are means of triplicates from three separate runs (*n* = 3). *p*-value for average HA concentration, CDW, and molecular weight in accordance with L-arabinose concentration was obtained using one-way ANOVA statistical analysis (α = 0.05). Culture conditions on modified 40-LBG-Y medium were pH 8.0, 250 rpm, and 25 °C. *p*-value = 0.0435. ^a^ HA concentration and CDW (g/L) values are presented as the mean ± SD.

## Data Availability

All data supporting the findings of this study are available within the article and its supplementary information files.

## Supplementary Materials

The following supporting information can be downloaded at: https://www.mdpi.com/article/10.3390/biom16060846/s1. Figure S1: Calibration curve for HA concentration as a function of absorbance at 600 nm. Figure S2: A normal plot of the standardized effects by a Plackett-Burman Design (PBD) is used to identify which factors significantly influence a response. Response is Response; α = 0.05. Figure S3: Residual plots for response to validate screening models by plotting the difference between observed and predicted response values. Figure S4: Comparative relative chromatogram of the wide distribution of HA molecular weight by recombinant *H. bluephagenesis* TD01 strains vs TD01-WT. Figure S5: GPC calibration curve for HA-*M_w_* by Standard polymer. Figure S6: Molecular weight distribution by effect of different concentrations of L-arabinose on the gene expression and Molecular weights of HA by *H. bluephagenesis* TD01-pMCSeSD-*araBAD-pmHasA*. Table S1. The primers list used in this study. Table S2: Fit Statistics of RSM. Table S3: Coefficients in terms of coded factors. Table S4: Final equation in terms of coded factors. Table S5: Final equation in terms of actual factors. Table S6. Comparative Relative peak of the wide distribution of HA molecular weight by recombinant *H. bluephagenesis* TD01 and TD01-WT. Table S7. Effect of different concentrations of L-arabinose on the gene expression and Molecular weights in the recombinant *H. bluephagenesis* TD01-pMCSeSD-*araBAD-pmHas*.
