# Supplementary material for: Engineering Halomonas bluephagenesis TD01 as a Robust Chassis for the Sustainable Production of Hyaluronic Acid
Source: Biomolecules. 2026 Jun 9;16(6):846. doi: 10.3390/biom16060846 (PMC13296553; doi:10.3390/biom16060846)
Supplement: Supplementary file 1 [file biomolecules-16-00846-s001.zip › biomolecules-4292289-supplementary.pdf]

Supplementary Materials

S1. List of the primers used in this study

Table S1. The primers list used in this study.

Genes fragments	Sequence (5` to 3`)	Source
00P20796	GGGTTATGCTAGTTAAAAAATGGTAACTTTTTACGGGTACC CATT	tsingke_00P20796
00P20797	AGGAGAATACTAGATGCCGATTTTTAAAAGACCCTGATCG	tsingke_00P20797
00P20798	TAAAAATCGCATCTAGTATTTCTCCTCTTTCTCTAGTACTGT	tsingke_00P20798
00P20799	TTACCATTTTTTAACTAGCATAACCCCTTGGGGC	tsingke_00P20799
00P20800	ATCACCCTGTAACTAGCATAACCCCTTGGGGCCTCTAAACG	tsingke_00P20800
00P20801	CAGGGTATTCATCTAGTATTTCTCCTCTTTCTCTAGTACTGTT	tsingke_00P20801
00P20802	GGAGAAATACTAGATGAATACCCTGAGCCAGGC	tsingke_00P20802
00P20803	GGTTATGCTAGTTACAGGGTGATGCGTTGATGATGAAC	tsingke_00P20803
00P20804	AAAACTGCTGTAACTAGCATAACCCCTTGGGGCC	tsingke_00P20804
00P20805	CAGGGTACGCATCTATTTCTCCTCTTTCTCTAGTACTG	tsingke_00P20805
00P20806	GAGAAATACTAGATGCGTACCCTGAAAAATCTGATTACC	tsingke_00P20806
00P20807	GGGTTATGCTAGTTACAGCAGTTTTTTACGGGTACCCCA	tsingke_00P20807
00P00652	CTGAGCTCTTCGATGCCGATTTTTAAAAAGACCCTGATC	tsingke_00P00652
00P00653	ATCGGCTCTTCAGCTTTAAAAAATGGTAACTTTTTACGGGTA CCCCA	tsingke_00P00653
00P58070	GCATGGTCTCACGCTCTGTTTCGTCCTCACGGA	tsingke_00P58070
00P58071	GCATGGTCTCTTACGTACTAGAGAAAGAGGAGAAATACTAG AT	tsingke_00P58071
00P58072	GCATGGTCTCAAGCGTTATGACAACTTGACGGCTAC	tsingke_00P58072
00P58073	GCATGGTCTCACGTAATGGAGAAACAGTAGAGAGTTG	tsingke_00P58073
00P58074	GCATGGTCTCGACGCCTGTTTCGTCCTCACGG	tsingke_00P58074
00P58075	GCATGGTCTCCGTACAGAGAAAGAGGAGAAATACTAGAT	tsingke_00P58075
00P58076	GCATGGTCTCGGCGTTTATGACAACTTGACGGCTAC	tsingke_00P58076
00P58077	GCATGGTCTCCGTACATGGAGAAACAGTAGAGAGTT	tsingke_00P58077
00P58078	GCATGGTCTCTATGCCTGTTTCGTCCTCACGG	tsingke_00P58078
00P58079	GCATGGTCTCACGTAAAAGAGGAGAAATACTAGATGCC	tsingke_00P58079
00P58080	GCATGGTCTCAGCATTTATGACAACTTGACGGC	tsingke_00P58080
00P58081	GCATGGTACTCTTACGATGGAGAAACAGTAGAGAGTT	tsingke_00P58081

S2. Standard curve for HA determination

For estimation of HA concentration in this study, A standard curve for Hyaluronic Acid (HA) was generated by plotting known concentrations of HA (typically ranging from 0.1 to 0.9 mg/ml depending on the assay) against their corresponding absorbance values, often measured using a 96-well microplate at a turbidimetric method by (Thermo MULTISKAN Spectrum, Thermo Fisher Scientific, USA).

Supplementary Materials

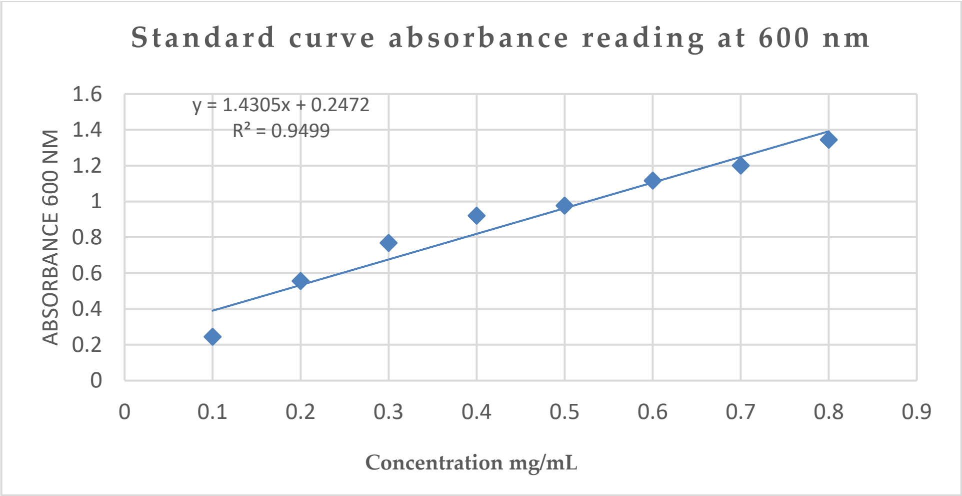

**Slope**	0.9709
**Intercept**	0.4194

Figure S1. Calibration curve for HA concentration as a function of absorbance at 600 nm.

S3. Plackett-Burman Design

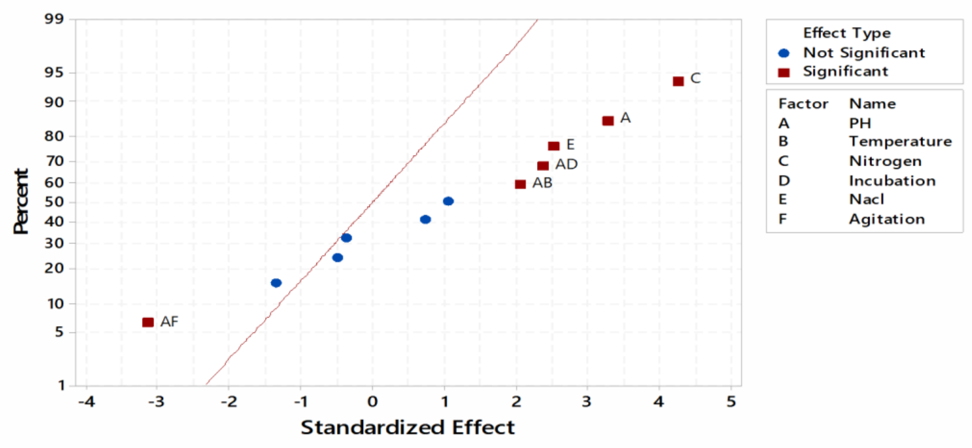

Figure S2. A normal plot of the standardized effects by a Plackett-Burman Design (PBD) is used to identify which factors significantly influence a response. Response is Response; $\alpha = 0.05$.

Supplementary Materials

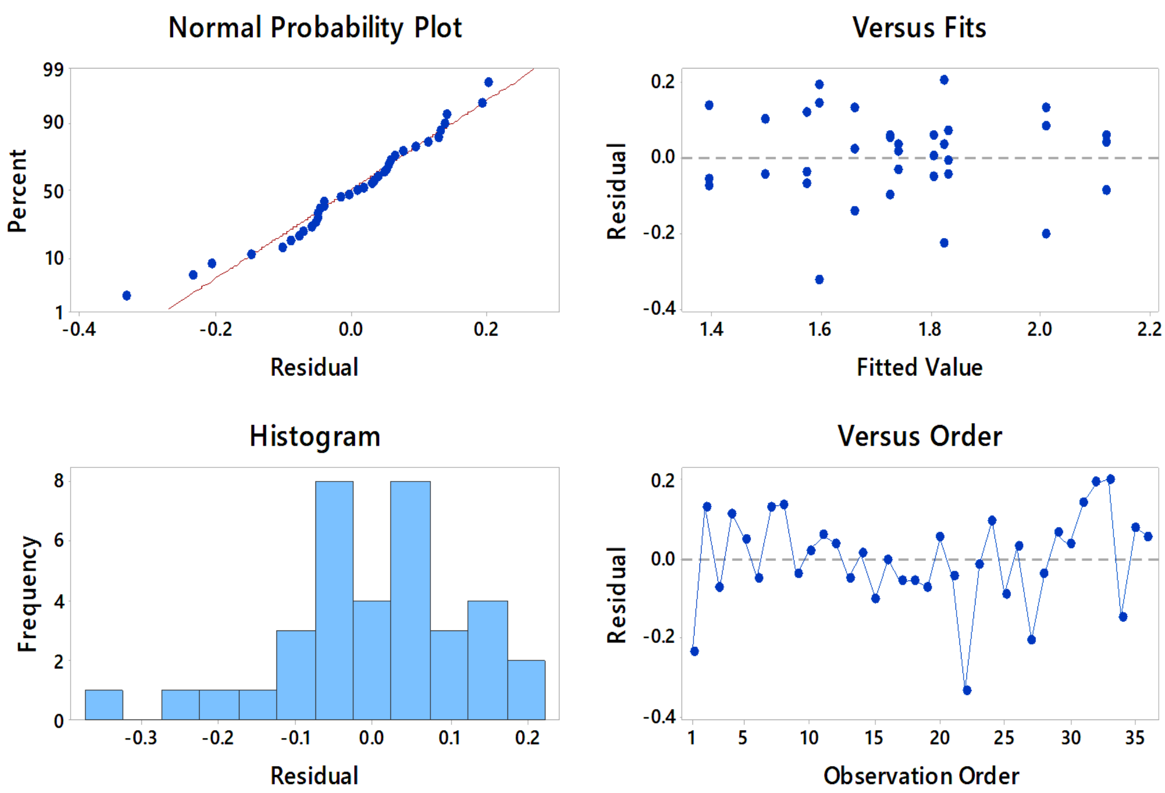

Figure S3. Residual plots for response to validate screening models by plotting the difference between observed and predicted response values.

S4. Response surface methodology

Table S2. Fit statistics of RSM.

**Std. Dev.**	**0.1258**	**R^2**	**0.9381**
Mean	1.82	Adjusted R^2	0.8825
C.V. %	6.92	Predicted R^2	0.6546
		Adeq Precision	14.2293

The discrepancy between the Predicted R^2 (0.6546) and Adjusted R^2 (0.8825) exceeds 0.2, suggesting potential model instability or data issues. While the Adeq Precision of 14.229 confirms an adequate signal-to-noise ratio for navigating the design space, you should investigate for outliers, consider response transformations, or perform model reduction. Finally, all empirical findings must be validated through confirmation runs.

Supplementary Materials

Table S3. Coefficients in terms of coded factors.

Factor	Coefficient Estimate	df	Standard Error	95% CI Low	95% CI High	VIF
Intercept	-5.43	1	3.31	-12.80	1.93	
A-A	-0.2727	1	0.4462	-1.27	0.7214	171.71
B-B	0.9697	1	0.4462	-0.0244	1.96	171.71
C-C	-9.32	1	3.32	-16.71	-1.92	380.06
AB	-0.0885	1	0.0445	-0.1876	0.0106	1.0000
AC	-0.1446	1	0.2224	-0.6402	0.3510	171.71
BC	0.4996	1	0.2224	0.0040	0.9952	171.71
A^2	-0.0808	1	0.0331	-0.1546	-0.0069	1.02
B^2	-0.0757	1	0.0331	-0.1496	-0.0019	1.02
C^2	-2.80	1	0.8286	-4.65	-0.9530	380.08

Coefficient estimates represent the expected change in response per unit factor change, holding others constant. In orthogonal designs, the intercept is the overall average, and coefficients are adjustments to it. Variance Inflation Factors (VIFs) measure multicollinearity; while 1 indicates perfect orthogonality, VIFs up to 10 are generally considered tolerable.

Table S4. Final equation in terms of coded factors.

R1	=
-5.43	
-0.2727	A
+0.9697	B
-9.32	C
-0.0885	AB
-0.1446	AC
+0.4996	BC
-0.0808	A^2
-0.0757	B^2
-2.80	C^2

The equation in terms of coded factors can be used to make predictions about the response for given levels of each factor. By default, the high levels of the factors are coded as +1, and the low levels are coded as -1. The coded equation is useful for identifying the relative impact of the factors by comparing the factor coefficients.

Table S5. Final equation in terms of actual factors.

R1	=
-4.96142	
+1.48613	A
+49.07868	B
+0.407871	C
-4.42508	A * B
-0.028914	A * C
+4.99587	B * C
-0.080796	A^2
-189.37181	B^2
-0.111973	C^2

The predictive equation in actual units allows for response estimation at specific factor levels. However, it should not be used to assess relative factor impact, as the coefficients are unit-dependent and the intercept is not centered in the design space.

S5. Molecular weight (Mw) determination of the non-induction plasmids without p_{araBAD} promoter.

Table S6. Comparative Relative peak of the wide distribution of HA molecular weight by recombinant *H. bluephagenesis* TD01 and TD01-WT.

SN	Strains	Mn (Dalton)	Mw (Dalton)	MP	Mz (Dalton)	Mz+1 (Dalton)	polydispersity	Mz/Mw
1	TD01-pmHasA	568800	1,1510,39	1490057	2027478	2937042	2.023626	1.761433
2	TD01-SezHasA	517044	1,059,027	2223742	1719633	2209271	2.048234	1.623786
3		5687	7528	4277	9977	12435	1.323587	1.325298
4	TD01-spHasA	595162	990,754	967629	1529165	2070661	1.664681	1.543435
5		5640	7192	4285	9240	11346	1.275122	1.284805
6	TD01-WT	252090	349728	419349	456959	569822	1.387313	1.306611
7		3074	3203	2954	3350	3516	1.042054	1.045912

Supplementary Materials

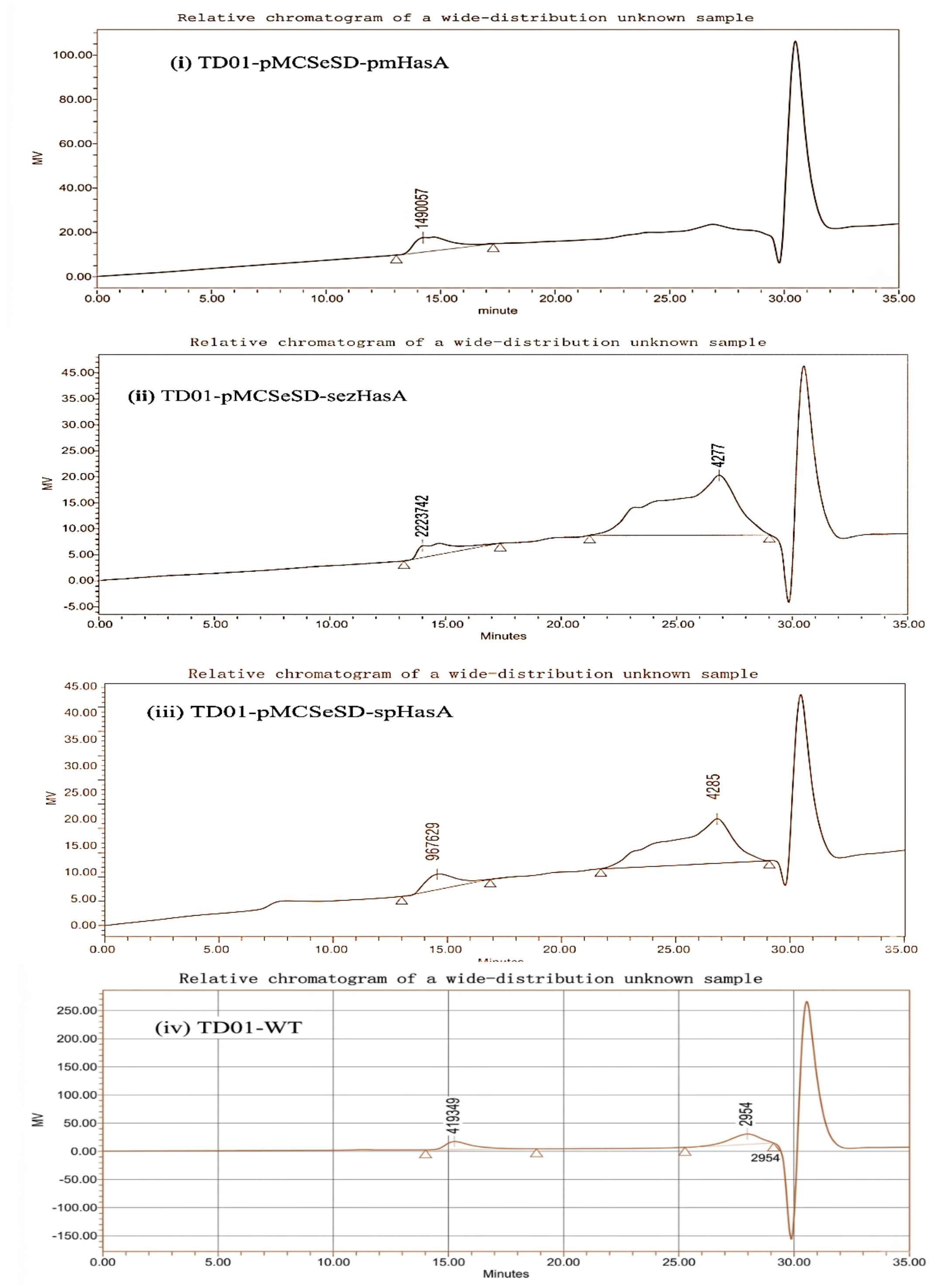

Figure S4. Comparative relative chromatogram of the wide distribution of HA molecular weight by recombinant *H. bluephagenesis* TD01 strains vs TD01-WT.

Supplementary Materials

S6. Effect of L-arabinose concentrations on the gene expression and Molecular weights (Mws) of HA, and the effect of the induction system on the H. bluephagenesis TD01-pMCSeSD-araBAD-pmHasA.

Table S7. Effect of different concentrations of L-arabinose on the gene expression and Molecular weights in the recombinant *H. bluephagenesis* TD01-pMCSeSD-*araBAD-pmHasA*.

Factors	Peak No.	Sample Name	Method	Retention time (min.)	Peak molar mass (g/mol)	Number Avg. molar mass (Mn) (g/mol)	Weight avg mass (g/mol) M_w (Daltons)	Z-avg. molar mass (g/mol)	Z+1-avg. molar mass (g/mol)	Visco. average molar mass (g/mol)	Polydispersity (PDI)
Control	1	HA-0	260320HA-SEC.PMX	12.842	2752459	2049722	**2,438,401**	2815509	31522611	2381382	1.19
	2			18.131	2574	1018	**3553**	10455	21770	2984	3.49
0.05%	3	HA-0.05		13.164	1654385	1012563	**2,043,548**	3109462	3933680	1886792	2.02
	4			18.438	1699	1860	**4417**	13615	262261	3703	2.37
0.1%	5	HA-0.1		12.426	4355900	2463978	**3564708**	4494145	5231683	3418515	1.45
	6			18.553	1455	1892	**8841**	61054	137031	6283	4.67
0.2%	7	HA-0.2		13.742	795204	531855	**1,549,380**	3661748	5345289	1316509	2.91
	8			18.908	1061	1050	**3545**	9472	14299	2933	3.38
0.4%	9	HA-0.4		12.163	6564716	3457907	**5,019,643**	6518804	7674094	4788683	1.45
	10			18.937	1017	1274	**5673**	18856	37046	4540	4.45
0.6%	11	HA-0.6		11.834	9841625	7974587	**9,212,013**	10413916	11513264	9030494	1.16
	12			16.793	14976	15973	**21735**	34382	53300	20441	1.36
0.8%	13	HA-0.8		12.238	7343107	5193672	**7,101,713**	9063410	10753937	6811208	1.37
	14			16.833	13989	2599	**12122**	30151	52871	10125	4.66
1%	15	HA-1		11.805	11962330	6225587	**9,674,869**	12982496	15613076	9171562	1.55
	16			16.420	25025	19794	**52120**	118911	185041	44978	2.63

Equation of HA-M_w calculation by GPC

Curve Fitting: Curve Fitting Equation:

$$y = -0.564707x + 13.648 \quad (1)$$

Curve Fitting Statistics: Residual Sum of Squares = 0.381609, Coefficient of Determination (R^2) = 0.983134, Linear Correlation Coefficient = -0.991523, Corrected Sum of Squares = 22.625363, and Standard Error of Y Estimate = 0.195348

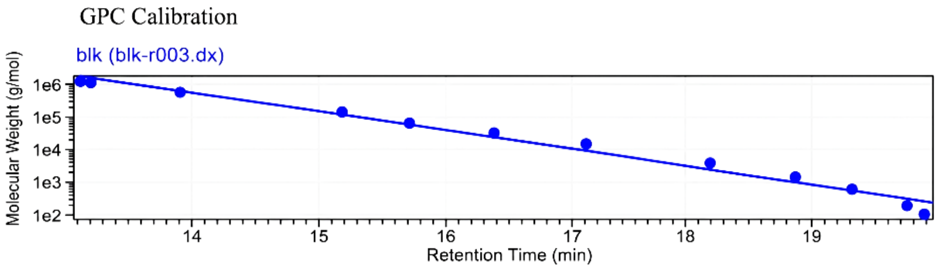

Figure S5. GPC calibration curve for HA-M_w by Standard polymer.

Supplementary Materials

(i) TD01-pMCSeSD-*AraBAD-pmHasA*

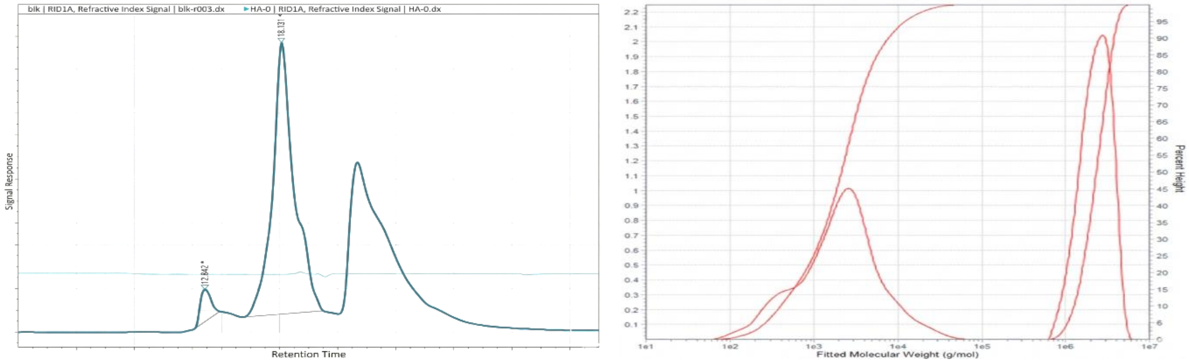

(ii) TD01-pMCSeSD-*AraBAD-pmHasA*, 0.05% L-arabinose

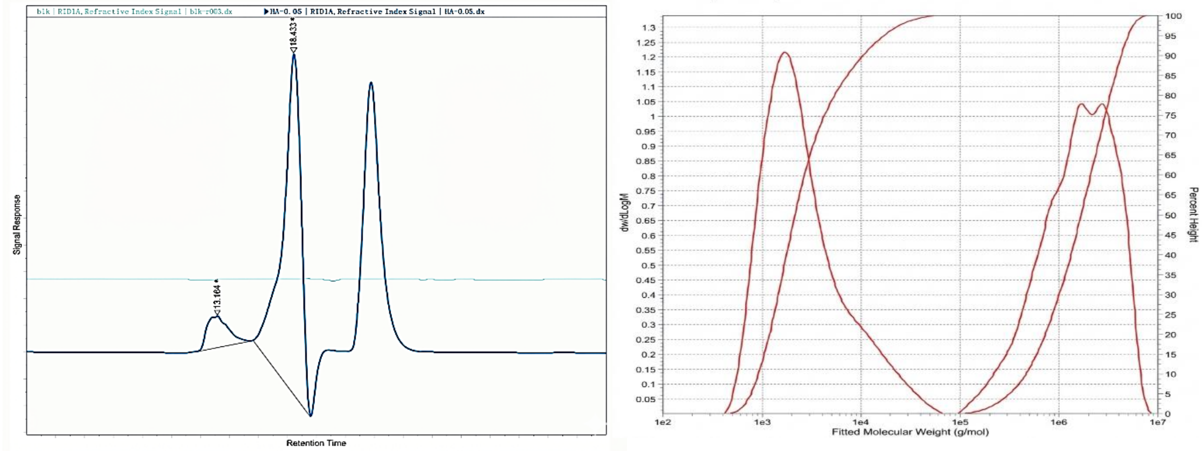

(iii) TD01-pMCseSD-*AraBAD-pmHasA*, 0.1% L-arabinose

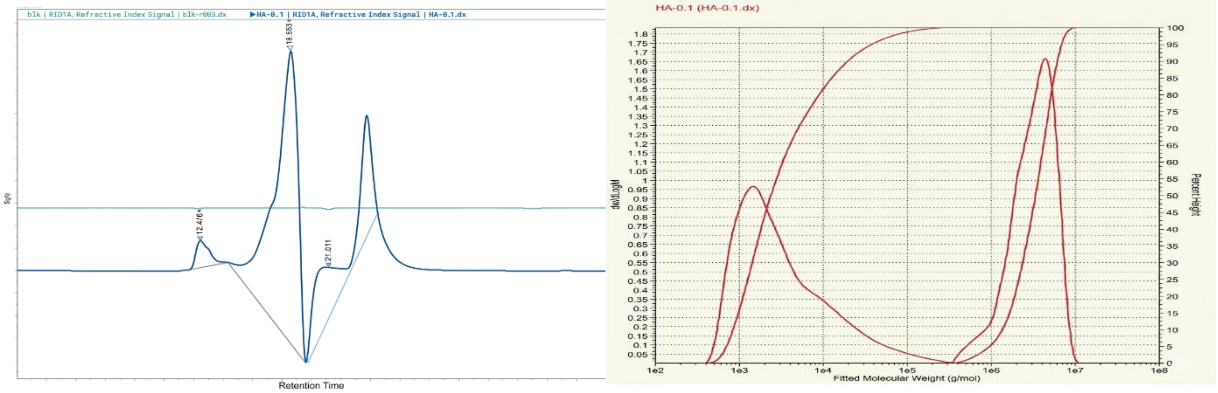

Supplementary Materials

(iv) TD01-pMCSeSD-*AraBAD-pmHasA*, 0.2% L-arabinose

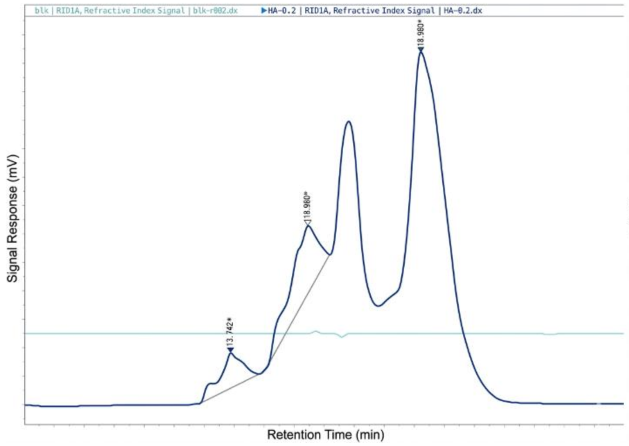

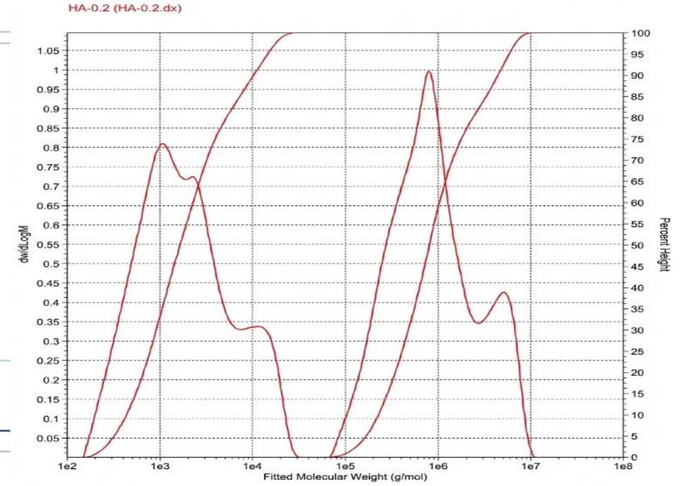

(v) TD01-pMCSeSD-*AraBAD-pmHasA*, 0.4% L-arabinose

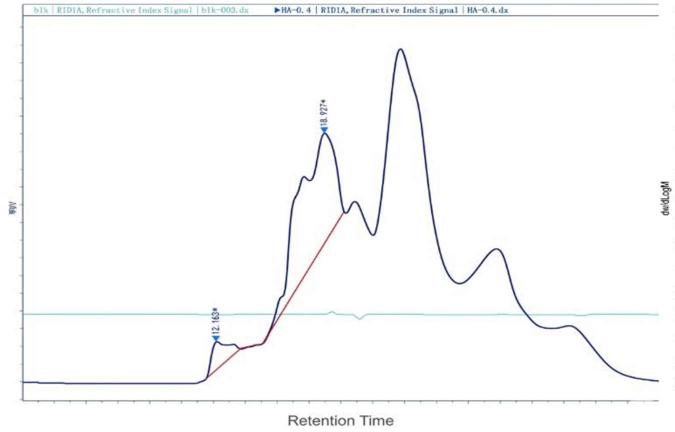

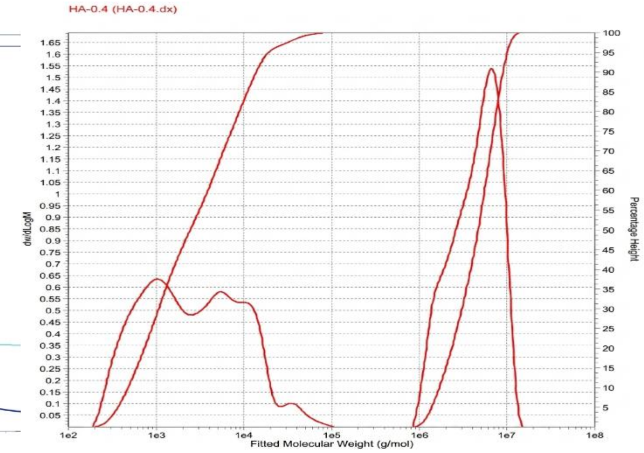

(vi) TD01-pMCSeSD-*AraBAD-pmHasA*, 0.6% L-arabinose

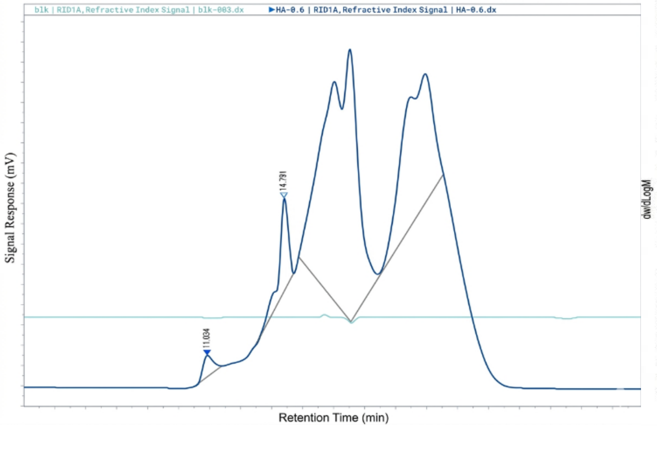

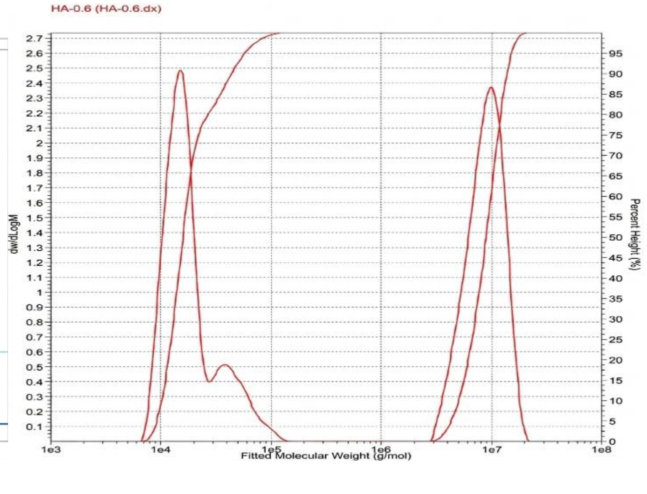

Supplementary Materials

(vii) TD01-pMCSeSD-*AraBAD-pmHasA*, 0.8% L-arabinose

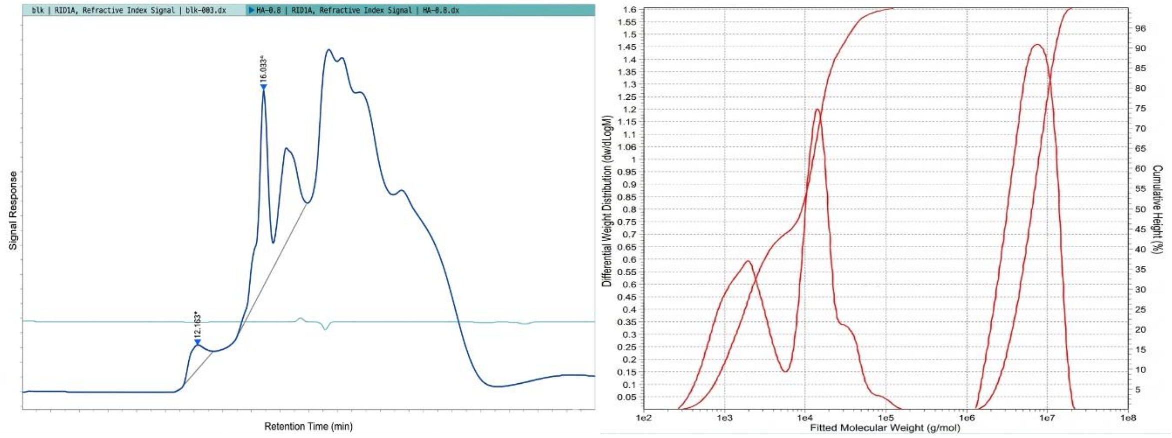

(viii) TD01-pMCSeSD-*AraBAD-pmHasA*, 1% L-arabinose

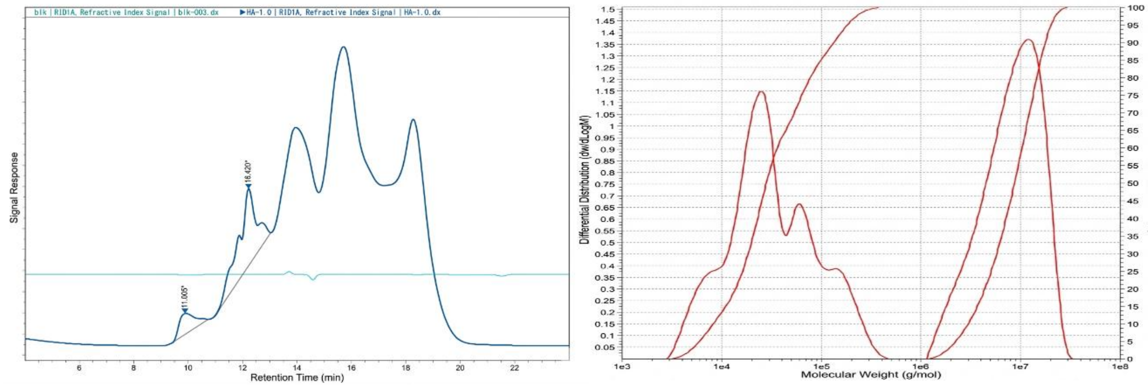

Figure S6. Molecular weight distribution by effect of different concentrations of L-arabinose on the gene expression and Molecular weights of HA by *H. bluephagenesis* TD01-pMCSeSD-*araBAD-pmHasA*.
